# Mingmu Xiaoyao granules regulate the PI3K/Akt/mTOR signaling pathway to reduce anxiety and depression and reverse retinal abnormalities in rats

**DOI:** 10.3389/fphar.2022.1003614

**Published:** 2022-10-05

**Authors:** Qiuyan Ma, Jian Zhou, Ziyi Yang, Yuxin Xue, Xinran Xie, Tiejun Li, Yingxin Yang

**Affiliations:** ^1^ Graduate School, Beijing University of Chinese Medicine, Beijing, China; ^2^ Ophthalmology Department, Beijing Hospital of Traditional Chinese Medicine, Capital Medical University, Beijing, China; ^3^ Ophthalmology Department, Dongfang Hospital, Beijing University of Chinese Medicine, Beijing, China; ^4^ Beijing Institute of Traditional Chinese Medicine, Beijing, China

**Keywords:** Mingmu Xiaoyao granules, retinal thickness, retinal blood flow, PI3K/Akt/mTOR pathway, autophagy, depression, chronic unpredictable mild stress, traditional Chinese medicine

## Abstract

**Objective:** To investigate the effects of Mingmu Xiaoyao granules (MMXY) on the morphology and function of the retina and the mechanism of PI3K/Akt/mTOR pathway-related proteins in rats with anxiety and depression induced by chronic unpredictable mild stress (CUMS).

**Methods:** Fifty-two male Sprague Dawley rats were randomly allocated to either a control (n = 14) or a simulated CUMS group (n = 38). The CUMS model was established successfully at 4 weeks. Six rats in each group were randomly selected to be sacrificed and their retinas isolated for histological examination. At 5 weeks, rats in the CUMS group were randomly allocated to the following groups: Model (CUMS + pure water), MMXY-H (CUMS + MMXY 7.2 g/kg/d), MMXY-L (CUMS + MMXY 3.6 g/kg/d), and CBZ (CUMS + Carbamazepine 20 mg/kg/d), with eight rats in each group. All rats were given the relevant intervention once a day. At 12 weeks, sucrose preference and open field tests were performed to evaluate the anxiety and depression status of rats. In live rats, optical coherence tomography angiography was used to measure retinal thickness and blood flow, while electroretinograms (ERGs) and visual evoked potentials (VEPs) were used to evaluate retinal function. The next day, the specimens were sacrificed for serological, histological, immunofluorescence, Western blot and transmission electron microscopy examinations to explore the mechanism of MMXY in CUMS rats.

**Results:** MMXY improved the anxiety and depression-like behavior of rats. Results of optical coherence tomography angiography showed that MMXY improved retinal inner thickness and blood flow in CUMS rats. MMXY improved the amplitude of a- and b-waves in the scotopic and photopic ERG, as well as N2 and P2 peak time and amplitude in the flash-VEP in CUMS rats. Retinal histological staining and transmission electron microscopy showed that MMXY reversed retinal morphology and ultrastructure in CUMS rats. MMXY reduced the expression of Beclin1 and LC3I/II proteins, regulated the PI3K/Akt/mTOR pathway, inhibited autophagy, and had a protective effect on the retina in CUMS rats.

**Conclusion:** MMXY may effectively improve retinal morphology and function as well as anxiety and depression-like behaviors in CUMS rats by regulating the PI3K/Akt/mTOR signaling pathway.

## Introduction

Clinically, in many patients, retinal diseases are associated with negative emotions, or even anxiety and/or depression. For instance, neovascular age-related macular degeneration is reported in 26.9% of patients with symptomatic depression and 25.5% with symptomatic anxiety (Fernandez-Vigo et al., 2021), and depressive symptoms are even more common than anxiety symptoms (Dawson et al., 2014). Among patients with diabetic retinopathy (DR), 25% suffer from depression and 13.5% suffer from anxiety (Xu et al., 2022). The incidence of DR is reduced after the use of antidepressants (Yekta et al., 2015). The number of patients with retinal vein occlusion (RVO) reportedly increased during and after the 2014 Football World Cup compared to the same period in 2013, and emotional stress was a risk factor (Lob et al., 2016). Visually impaired patients with retinitis pigmentosa all present mild to moderate depression (Moschos et al., 2015) and patients with glaucoma are at risk of depression and/or anxiety, women being at higher risk than men (Chen et al., 2018; Abe et al., 2021). Therefore, many retinal diseases are closely related to psychosomatic diseases.

The classic method used to induce anxiety and depression is chronic unpredictable mild stress (CUMS) (Willner, 2005). In general, unpredictable psychological stressors are more likely to promote depressive-like behaviors than predictable ones (McEwen, 2000). CUMS-induced anxiety and depressive behaviors in rats can affect brain regions such as the frontal cortex (Yu et al., 2021) and hippocampus (Zhuo et al., 2020) by regulating autophagy. Autophagy is a lysosomal mediated degradation process that removes misfolded proteins and excess or damaged organelles, restores intracellular nutrients, defends against pathogens, and plays a key role in homeostasis of the intracellular environment (Patel et al., 2013; Jiang et al., 2018). The PI3K/Akt/mTOR signaling pathway is a common pathway in autophagy, and plays an important role in regulating cell survival, differentiation, proliferation and migration (Yu and Cui, 2016).

Mingmu Xiaoyao granule (MMXY) is a traditional Chinese medicine (TCM) supplemented with liver-soothing and mingmu herbs on the basis of Xiaoyao Powder, and is widely used in patients with retinal diseases complicated with liver depression symptoms (Yang et al., 2012). The symptoms of liver depression in TCM include depression, irritability, loss of appetite, and others, which are similar to the symptoms of anxiety and depression recognized by Western medicine (Wang et al., 2019). Numerous studies have confirmed that drugs can regulate the PI3K/Akt/mTOR autophagy signaling pathway, and ultimately improve a variety of retinal diseases such as DR, glaucoma and age-related macular degeneration. By modulating this pathway, mangiferin inhibits angiogenesis of rat retinal capillary endothelial cells in diabetic rats (Shi et al., 2021), ligustrazine has a protective effect on experimental glaucoma (Du et al., 2021) and doxazosin inhibits the angiogenesis of choroidal neovasculogenesis and reduces the occurrence of age-related macular degeneration (Guo et al., 2017). However, it is not clear whether MMXY regulation of the pathway can improve CUMS-induced anxiety and depression-like behavior and retinal function in rats.

On the basis of previous experiments and existing literature, we established a CUMS rat model to investigate whether MMXY can improve anxiety and depression-like behavior and protect rat retina by regulating the PI3K/Akt/mTOR signaling pathway. If so, MMXY may provide a good therapeutic strategy for patients with retinal disease who have anxiety and depression-like symptoms.

## Materials and methods

### Animals and groups

Fifty-two healthy 8-week-old male Sprague Dawley rats, weighing 200 ± 20 g, were purchased from SPF Biotechnology Co., Ltd (Beijing, China). This study was conducted in strict accordance with the Guide for the Care and Use of Laboratory Animals (NIH Publication No. 85-23,1996). The experimental procedure was approved by the local ethics committee (reference 2020070101). The rats were housed in a specific-pathogen-free facility under artificially controlled conditions (temperature, 20°C–24°C; relative humidity, 50%–60%; light/dark cycle 12/12 h).

**FIGURE 1:**
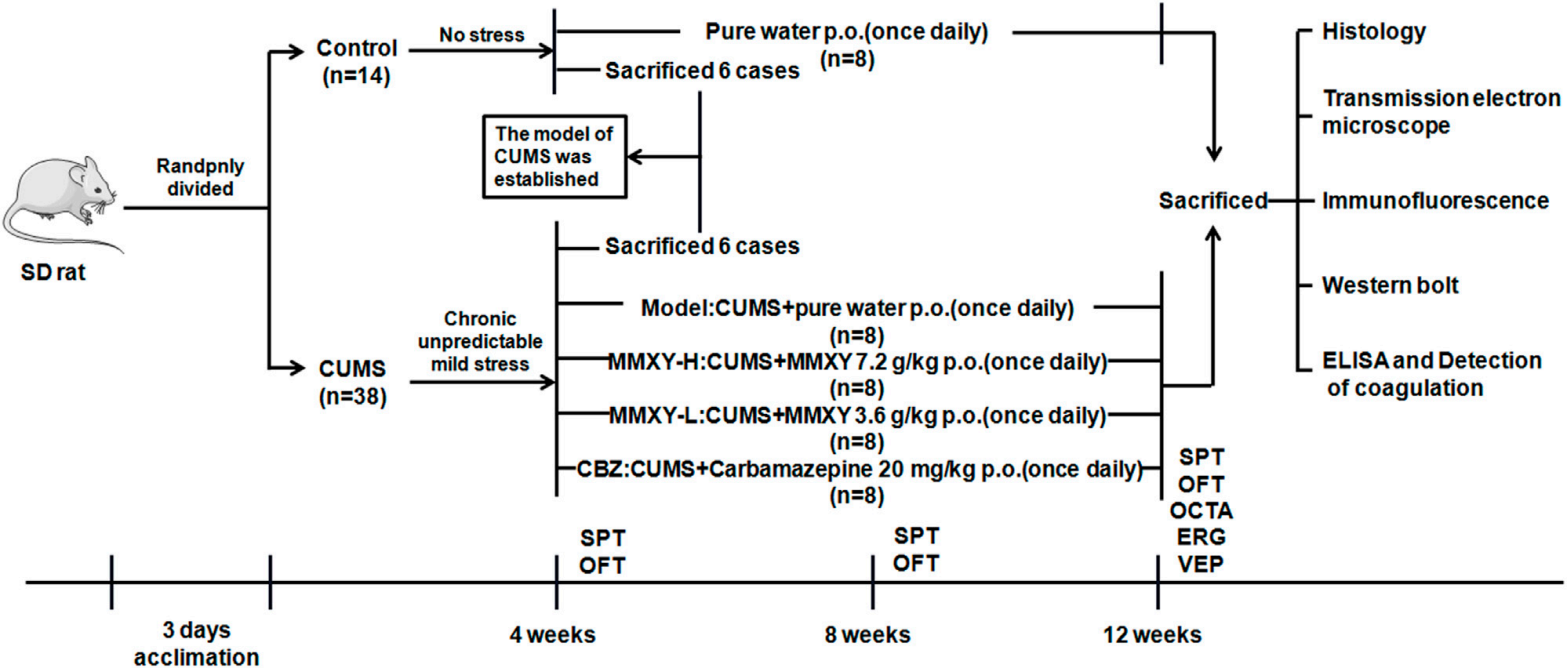
Experimental procedure of MMXY intervention study. The model was successfully established in week 4, and then CUMS group was randomly divided into Model group, MMXY-H group, MMXY-L and CBZ groups. Rats in each group were given drugs or distilled water lavage, once a day, until 12 weeks of death for sampling. SPT, sucrose preference test; OFT, open field test; OCTA, optical coherence tomography angiography; ERG, electroretinogram; VEP, visual evoked potential.

The rats were randomly allocated to control (n = 14) and CUMS groups (n = 38). Rats in the control group were given a conventional diet and normal water, and the rats in the CUMS group were given modeling stimulation for 4 weeks. The results of sucrose preference (SPT) and open field (OFT) tests showed that the CUMS model was established successfully. Six rats randomly selected from each group were deeply anesthetized with chloral hydrate (0.4 g/kg Body Weight), and then their retinas were collected for histological examination. From the fifth week, the remaining rats in the CUMS group (n = 32) were randomly allocated to receive CUMS + pure water (model group), CUMS + MMXY 7.2 g/Kg/d (MMXY-H group), CUMS + MMXY 3.6 g/Kg/d (MMXY-L group) or CUMS + Carbamazepine 20 mg/Kg/d (CBZ group), with eight rats included in each group. All rats were given the relevant intervention by lavage once a day. Each rat’s body weight was recorded every Friday during the experiment, and SPT and OFT were performed every 4 weeks (Figure 1).

### Drugs

Mingmu Xiaoyao granules were composed of 10 traditional Chinese medicines: Bupleurum chinenseDC [Umbelliferae; Bupleuri radix], Angelica sinensis (Oliv.) Diels [Umbelliferae; Angelicae sinensis radix], Paeonia lactiflora Pall [Ranunculaceae; Paeoniae radix alba], *Glycyrrhiza* uralensis Fisch. [Leguminosae; Glycyrrhizae radix et rhizoma], Poria cocos (Schw.)Wol£ [Polyporaceae; Poria], Atractylodes macrocephala Koidz [Compositae; Atractylodis macrocephalae rhizoma], *Chrysanthemum* morifolium Ramat. [Compositae; Chrysanthemi flos], Lycium barbarum L [Solanaceae; Lycii fructus], Acorus tatarinowii Schott [Araceae; Acori tatarinowii rhizoma], Paeonia suffruticosa Andr. [Ranunculaceae; Moutan cortex]. The ratio was 3:3:3:5:3:2:3:3:3:3.

For the above ten herbs, Angelica sinensis (Oliv.) Diels, Acorus tatarinowii Schott, and Paeonia suffruticosa Andr were steamed with eight times the amount of water for 8 h, and the volatile oil was collected for later use. The distilled aqueous solution was also collected in another container for later use. The medicinal residues and the other seven herbs were boiled twice in water for 2 h and 1 h, respectively. Ten times the amount of water was added for each boiling. The decoction was filtered, and the filtrate was combined with the above-mentioned aqueous solution and concentrated into a thick paste with a relative density of 1.25–1.30. Then dry under reduced pressure and pulverize. Add an appropriate amount of dextrin for a mixture, and granulate with ethanol later. The granules were dried, sprayed with volatile oil, mixed well, and prepared to obtain MMXY granules. One Gram of fine powder of MMXY was obtained from 3.03 g of raw herbs. The granules were provided by the Traditional Chinese Medicine Pharmacy of Guang anmen Hospital, China Academy of Traditional Chinese Medicine (batch number Z20063185, Beijing, China). The phytoconstituents of MMXY were analyzed by UPLC-MS (Vanquish, Thermo Fisher Scientific, MA, United States). We matched the detected substances in Yet another Traditional Chinese Medicine database, based on the name of the substances and InChIKey number. The MMXY-high (MMXY-H) and -low (MMXY-L) doses were 7.2 and 3.6 g/kg/d (the latter a clinically effective dose in humans), respectively (Xu et al., 2006).

The dosage of Carbamazepine (CBZ) was 20 mg/Kg/d in accordance with the drug instructions (batch number Sinopharmed H11022279, Beijing, China) and literature (Naghizadeh et al., 2016; Elsherbiny et al., 2019), using 8‰ sodium carboxymethyl cellulose to prepare 2.8 mg/ml solution. Carbamazepine powder (batch number RH328280, R012551-25G) and sodium carboxymethyl cellulose (R008133-100G, CAS number 9004-32-4) were purchased from RON Company (Shanghai, China). Compound tropicamide eye drops including tropicamide 5 mg/ml and norepinephrine hydrochloride 5 mg/ml, and Oxybuprocaine hydrochloride eye drops (4 mg/ml) were purchased from Santen Pharmaceutical (Jiangsu, China) Co., Ltd. Carboxymethyl Cellulose Sodium Eye Drops (1%, 4 mg/0.4 ml) were purchased from Allergan (County Mayo, Ireland).

### Reagents

Hematoxylin and eosin (HE) (batch number C211201, C220307) were purchased from BaSo (Zhuhai, China). FAS eyeball fixator was purchased from Service Bio (Wuhan, China). Gluta Fixator (electron microscopy only, 4%), BCA protein assay kit and Rainbow 245 Broad Spectrum Protein Marker were purchased from Solarbio (Beijing, China). Dopamine (DA) Kit (orb410818) was purchased from Biorbyt (Cambridge, United Kingdom). Antibodies for Akt (2920S), Phospho-Akt (Ser473) (4060s), mTOR (2983s), and Phospho-mTOR (Ser2448) (5536T) were purchased from Cell Signaling Technology (Danvers, MA, United States). LC3A/LC3B (PA1-16931) antibody was purchased from Thermo (Waltham, MA, United States). Anti-rabbit IgG (H + L) antibody (DyLight 680-labeled), and anti-mouse IgG (H + L) antibody (DyLight 800-labeled) were purchased from Sera Care (KPL) (Milford, United States). Corticosterone (CORT) Kit (ab108821), GAPDH (ab8245), PI3Kp85α (ab227204), Phospho-PI3Kp85 (Y607) (ab182651), Phospho-Akt (Thr308) (ab38449), Beclin1 (ab62557), goat anti-rabbit IgG H&L (Alexa Fluor^®^ 488) (ab150077), and mounting medium with DAPI-aqueous (ab104139) were purchased from Abcam (Cambridge, United Kingdom).

### UPLC-MS/MS analysis of MMXY

LC-MS/MS analysis was performed on a UHPLC system (Vanquish, Thermo Fisher Scientific) with a Waters UPLC BEH C18 column (1.7 μm × 2.1 mm × 100 mm). The flow rate was set at 0.5 ml/min and the sample injection volume was set at 5 μl. The mobile phase consisted of 0.1% formic acid in water (A) and 0.1% formic acid in acetonitrile (B). The multi-step linear elution gradient program was as follows: 0–11 min, 85%–25% A; 11–12 min, 25%–2% A; 12–14 min, 2%–2% A; 14–14.1 min, 2%–85% A.

An Orbitrap Exploris 120 mass spectrometer coupled with Xcalibur software was employed to obtain the MS and MS/MS data based on the IDA acquisition mode. During each acquisition cycle, the mass range was from 100 to 1,500, the top four of every cycle were screened and the corresponding MS/MS data were further acquired. Settings were as follows: sheath gas flow rate 35 Arb; aux gas flow rate 15 Arb; ion transfer tube temperature 350°C; vaporizer temperature 350°C; full MS resolution 60,000; MS/MS resolution 15,000; collision energy 16/38/42 in NCE mode; spray voltage 5.5 kV (positive) or −4 kV (negative).

### CUMS program stimulation in rats

The process used in our model is widely established in rodents, which uses a variety of unpredictable stressors to induce anxious and depressive-like behaviours seen in humans (Shang et al., 2017). The CUMS rats were subjected to modeling stimulation slightly modified from that described by Willner (Willner, 2005), including tail-clamping stimulation (1 min), binding (4–6 h), warm stimulation (5 min), fasting (24 h), water prohibition (24 h), fouling cage stimulation (24 h), and no padding stimulation (24 h). The stimuli were randomly assigned each day, so the animals could not predict the stimulus.

### Sucrose preference test

The SPT is the gold standard for verifying lack of pleasure in laboratory animals (Willner et al., 1987). Rats were fed in a single cage after 24 h without water. They were given a bottle of pure water and a bottle of 1% sucrose solution, each randomly located in the cage. The bottles were weighed before and 1 h after they were given to the rats. The sucrose preference was calculated using the following equation: sucrose preference (%) = sucrose intake/(sucrose intake + pure water intake) × 100.

### Open field test

The OFT was designed by Hall (1934) to quantify the anxiety and depression behavior of rats (Zimcikova et al., 2017). The experiment was carried out in a 100 cm × 100 cm × 40 cm open-lid box, which was divided into 25 squares at the bottom. We fixed the camera on top of the box to capture the whole box. To avoid the influence of external sound on rats’ behavior, the box was placed in a soundproof environment during the experiment. A rat was placed alone in the center of the box and allowed to roam free for 5 min on a timer. After this time, clean the urine and feces from the box and disinfect the box with 75% alcohol. Once the box dried, the next rat experiment began. Central grid residence time, horizontal activity, vertical activity and grooming times of the rats were recorded.

### Optical coherence tomography angiography imaging of the retina

A swept source-OCTA system (VG200D, SVision Imaging, Ltd., Henan, China) was used to measure retinal thickness and blood flow. The rats were anesthetized by intraperitoneal injection of chloral hydrate (0.4 g/kgBW), compound tropicamide eye drops were used to dilate pupils, oxybuprocaine hydrochloride eye drops were used for ocular surface anesthesia, and carboxymethyl cellulose sodium eye drops were used to keep eyes moist. The images of rat retina were centered on the optic disc. The thickness and blood flow of each retinal layer were automatically measured using built-in software. The measurement range was a 9 mm diameter area centered on the optic disc.

### Electroretinogram and visual evoked potential

The ERG is an important examination method to evaluate retinal function (Kremers and Tanimoto, 2018). ERGs of live rats were recorded using the Roland RETI-Port/scan21 (Brandenburg, Germany) in a professional dark and soundproof ERG laboratory. Rats were adapted to the dark environment for 24 h. Pupils were dilated, ocular surface anesthetized and moistened as described for OCTA. The active electrode was a ring electrode placed on the cornea, the reference and ground electrodes were placed in the subcutaneous space of the cheek and the tail. Waveforms were recorded with a white LED light against a dark background with a duration of 5 ms and a luminance range of 0.01–10.0 cd s m^−2^. According to the International Society for Clinical Electrophysiology of Vision Standard (Robson et al., 2022), Scotopic 0.01 ERG, Scotopic 3.0 ERG, Scotopic 10.0 ERG and Scotopic 3.0 Oscillatory Potential ERG were examined in order. After 10 min of bright adaptation, Photopic 3.0 ERG and Photopic 3.0 Flicker 30 Hz ERG were performed with 3.0 cd s m^−2^ intensity light source under bright background for 250 ms. Three stable waveforms were recorded for each animal.

Visual evoked potentials (VEPs) can provide important diagnostic information regarding the functional integrity of the visual system (Odom et al., 2016). The needle recording electrode was placed under the skin of the skull midline anterior to the occipital protuberance, the reference and ground electrodes were placed in the subcutaneous space of the cheek and the tail. Unexamined eyes were covered with an opaque cloth to avoid interference. The intensity of the optical stimulator was 3.0 cd s m^−2^; the background light intensity was 30 cd s m^−2^; flash rate of 1.0 and 12 Hz, each item was superimposed 30 times.

### Tissue sample collection

At 12 weeks, the rats were anesthetized by intraperitoneal injection of chloral hydrate (0.4 g/kgBW). Blood was taken from the abdominal aorta. 2 ml of blood placed in tubes without EDTA and centrifuged at 3,500 rpm for 15 min. Then the serum was collected and separated into EP tubes and stored at −80°C for later use. The remaining blood was placed into a tube containing sodium citrate anticoagulant, to conduct the blood coagulation test as soon as possible. The eyeballs were dissected on an ice plate and retinal tissues were detached, then the eyeballs were fixed with FAS (eyeball fixator) and Gluta (electron microscope fixator). The retinal tissues were frozen with liquid nitrogen and stored at −80°C.

### Determination of blood coagulation levels

The levels of activated partial thromboplastin time (APTT), prothrombin time (PT) and plasma fibrinogen (FIB) were measured by automatic hemagglutination apparatus ACL-TOP 700 LAS (Werfen, MA, United States).

### Determination of CORT and DA levels

Serum CORT and DA levels were measured. According to the standard concentration and O.D. values of CORT, and the standard concentration and corrected O.D. values of DA (readings at 540 nm were subtracted from the readings at 450 nm), a four-parameter logistic curve-fit with a high degree of fit was calculated. The O.D. and the corrected O.D. values were substituted into the curve equation to calculate the serum concentration.

### Histology

The eyeballs were fixed in FAS eyeball fixator for 24 h. Paraffin-embedded eyeballs were cut into 3-μm-thick slices and stained with HE. A panoramic SCAN scanner (3D histech, Budapest, Hungary) was used to scan images and CaseViewer software was used to analyze them. We take 6-8 points from different locations of the retina to measure the thickness of each layer and count the number of outer nuclear layer (ONL) and inner nuclear layer (INL).

### Transmission electron microscope

The retinas were detached and fixed in Gluta at 4°C overnight. The next day, the retinal tissue was sliced into 1-mm^2^ sections, dehydrated with acetone, and embedded with Spon 812 embedding agent (SPIZB-S0060, PA, United States). The sections were cut into 70-nm-thick slices with an EM UC6 slicer (Leica, Wetzlar, Germany), and then slices were fixed to a copper wire and stained with uranium acetate and citric acid each for 30 min. Transmission electron microscope (TEM) images of the retina were captured and analyzed using a Hitachi H-7650 electron microscope (Hitachi, Tokyo, Japan).

### Immunofluorescent staining

Paraffin-embedded eyeballs were cut into 3-μm-thick slices, which were dewaxed with xylene, hydrated with graded ethanol, heated with sodium citrate water bath and then sealed with serum. The primary antibody was incubated overnight, and the second antibody IgG (ab150077) was incubated for 1 h on the second day. After washing three times with phosphate buffer saline, Mounting Medium (ab104139) was added to the slices, which were sealed with a cover slip and observed with a confocal laser scanning microscope (Zeiss LSM710, Oberkochen, Germany). Images were taken at an excitation wavelength of 488 nm and analyzed using ZEN Version 3.5.

### Western blot analysis

Total retinal protein was extracted using protein lysis buffer and quantified using a BCA protein assay kit. After denaturation at 95°C for 10 min, equivalent amounts of protein from each sample were separated by 6%–15% SDS–PAGE and transferred onto polyvinylidene fluoride membranes. This was then sealed with Tris Buffered Saline with Tween-20 containing 5% fat-free milk for 1 h at 37°C. The membranes were incubated with primary antibody overnight at 4°C, then incubated with corresponding fluorescent secondary antibody IgG (5230-0402, 5230-0415) for 1 h at 37°C. The blots were detected using an Odyssey Bicolor Infrared Fluorescence imaging system (LI-COR, NE, United States). The comprehensive density was quantified using ImageJ (National Institutes of Health, Bethesda, MD, United States). The GAPDH antibody was used to confirm equal protein load in each lane.

### Statistical analysis

All statistical analyses were performed using SPSS 22.0 software. Shapiro-wilk test was used to test normality and F-test was used to test homogeneity of variance. When the distribution was normal and the variances were equal, one-way analysis of variance was used, multiple comparisons were corrected using Fisher’s least significant difference test, and data were described by mean ± standard deviation. If the data did not follow a normal distribution or variance was inconsistent, a nonparametric test (Kruskal–Wallis rank-sum test) was applied, followed by Dunnett’s test to analyze significant differences between groups, with the median (inter-quartile range) describing the data. Pearson test was used for correlation analysis. *p* < 0.05 was considered statistically significant.

## Results

### Identification of main components of MMXY

**FIGURE 2:**
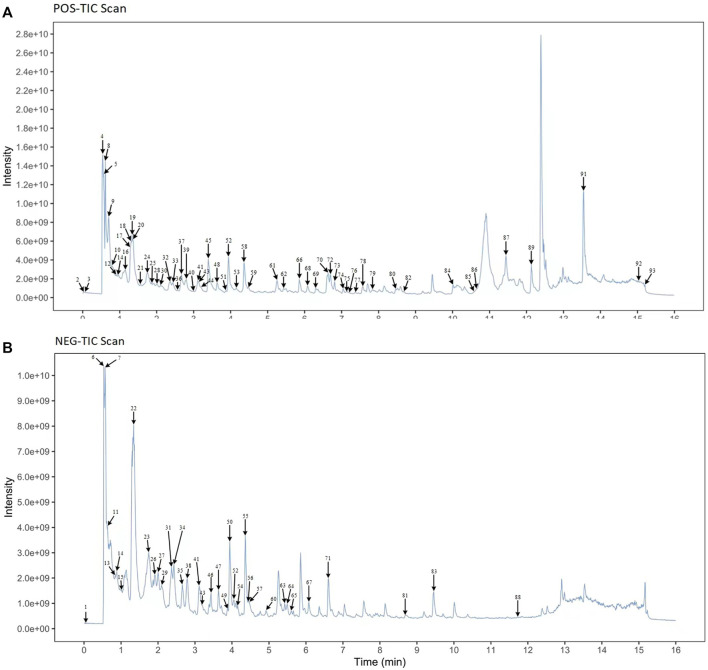
**(A)** Mass spectrometry of sample solutions in positive ion mode. **(B)** Mass spectrometry of sample solutions in negative ion mode.

UPLC-MS/MS techniques were used to determine the main components of MMXY. Figure 2 shows the UPLC-MS/MS total ion flow diagram of MMXY sample solution. Table 1 shows the identification and analysis of chemical components in MMXY sample solution.

**TABLE 1:** Ingredients of MMXY.

No.	Retention time median (t/min)	Ion	Mass charge ratio median (m/z)	Chemical formula	Compounds name	InChIKey	Plant origin of the compounds
1	0.0294	M-H	255.233	C_16_H_32_O_2_	Palmitic Acid	IPCSVZSSVZVIGE-UHFFFAOYSA-N	Bupleurum chinenseDC, Angelica sinensis (Oliv.) Diels, Poria cocos (Schw.)Wol£, Atractylodes macrocephala Koidz, *Chrysanthemum* morifolium Ramat, Lycium barbarum L, Acorus tatarinowii Schott
2	0.0328	M + H	153.127	C_10_H_16_O	(R)-Campholenic aldehyde	OGCGGWYLHSJRFY-UHFFFAOYSA-N	Bupleurum chinenseDC, *Chrysanthemum* morifolium Ramat
3	0.0622	M + H	139.075	C_8_H_10_O_2_	2-Methoxy-4-methylphenol	PETRWTHZSKVLRE-UHFFFAOYSA-N	Paeonia lactiflora Pall
4	0.5506	M + H	104.107	C_5_H_14_NO	Choline chloride	OEYIOHPDSNJKLS-UHFFFAOYSA-N	Angelica sinensis (Oliv.) Diels, Poria cocos (Schw.)Wol£
5	0.5604	M + H	136.061	C_5_H_5_N_5_	Adenine	GFFGJBXGBJISGV-UHFFFAOYSA-N	Angelica sinensis (Oliv.) Diels, Poria cocos (Schw.)Wol£, *Chrysanthemum* morifolium Ramat
6	0.611	M-H	117.019	C_4_H_6_O_4_	Succinis acid	KDYFGRWQOYBRFD-UHFFFAOYSA-N	Angelica sinensis (Oliv.) Diels
7	0.6251	M-H	125.024	C_6_H_6_O_3_	Pyrogallol	WQGWDDDVZFFDIG-UHFFFAOYSA-N	Paeonia lactiflora Pall
8	0.6626	M + H	124.039	C_6_H_5_NO_2_	Nicotinic acid	PVNIIMVLHYAWGP-UHFFFAOYSA-N	Angelica sinensis (Oliv.) Diels, Lycium barbarum L
9	0.6724	M + H	166.086	C_9_H_11_NO_2_	Phenylalanine	COLNVLDHVKWLRT-UHFFFAOYSA-N	Atractylodes macrocephala Koidz
10	0.7147	M + H	355.102	C_16_H_18_O_9_	1-O-caffeoylquinic acid	CWVRJTMFETXNAD-DUXPYHPUSA-N	*Chrysanthemum* morifolium Ramat
11	0.7163	M-H	183.03	C_8_H_8_O_5_	Methylgallate	FBSFWRHWHYMIOG-UHFFFAOYSA-N	Paeonia lactiflora Pall
12	0.8108	M + H	195.065	C_10_H_10_O_4_	Isoferulic acid	QURCVMIEKCOAJU-HWKANZROSA-N	Bupleurum chinenseDC
13	0.919	M-H	179.035	C_9_H_8_O_4_	Caffeic acid	QAIPRVGONGVQAS-UHFFFAOYSA-N	*Chrysanthemum* morifolium Ramat, Paeonia suffruticosa Andr
14	0.9271	M-H	563.142	C_26_H_28_O_14_	5,7-dihydroxy-2-(4-hydroxyphenyl)-8-[3,4,5-trihydroxy-6-(hydroxymethyl)oxan-2-yl]-6-(3,4,5-trihydroxyoxan-2-yl)chromen-4-one	OVMFOVNOXASTPA-UHFFFAOYSA-N	*Glycyrrhiza* uralensis Fisch
15	0.9622	M_H	151.061	C_5_H_12_O_5_	L-Arabitol	HEBKCHPVOIAQTA-UHFFFAOYSA-N	Bupleurum chinenseDC
16	1.3322	M + H	123.08	C_8_H_10_O	3-Ethylphenol	HMNKTRSOROOSPP-UHFFFAOYSA-N	Angelica sinensis (Oliv.) Diels
17	1.3328	M + H	105.07	C_8_H_10_O	2-Phenylethanol	WRMNZCZEMHIOCP-UHFFFAOYSA-N	Angelica sinensis (Oliv.) Diels
18	1.3328	M + H	135.08	C_9_H_10_O	2,4-Dimethylbenzaldehyde	GISVICWQYMUPJF-UHFFFAOYSA-N	Angelica sinensis (Oliv.) Diels
19	1.3421	M + H	463.16	C_23_H_26_O_10_	Methylnissolin-3-O-glucoside	PCIXSTFFMHVOMF-UHFFFAOYSA-N	Angelica sinensis (Oliv.) Diels
20	1.4398	M + H	611.159	C_27_H_30_O_16_	Rutin	IKGXIBQEEMLURG-UHFFFAOYSA-N	Bupleurum chinenseDC, *Glycyrrhiza* uralensis Fisch Lycium barbarum L
21	1.4424	M + H	303.05	C_15_H_10_O_7_	Quercetin	REFJWTPEDVJJIY-UHFFFAOYSA-N	Bupleurum chinenseDC, Poria cocos (Schw.)Wol£, *Chrysanthemum* morifolium Ramat, Paeonia suffruticosa Andr
22	1.4726	M-H	433.113	C_21_H_22_O_10_	Flavanone + 3O, O-Hex	DLIKSSGEMUFQOK-UHFFFAOYSA-N	*Glycyrrhiza* uralensis Fisch
23	1.6781	M-H	417.119	C_21_H_22_O_9_	Liquiritin	DEMKZLAVQYISIA-UHFFFAOYSA-N	*Glycyrrhiza* uralensis Fisch
24	1.6986	M + H	193.049	C_10_H_8_O_4_	Scopoletin	RODXRVNMMDRFIK-UHFFFAOYSA-N	Bupleurum chinenseDC, Angelica sinensis (Oliv.) Diels, *Glycyrrhiza* uralensis Fisch, Atractylodes macrocephala Koidz, Lycium barbarum L
25	1.929	M + H	207.065	C_11_H_10_O_4_	Citropten	NXJCRELRQHZBQA-UHFFFAOYSA-N	Bupleurum chinenseDC
26	1.9686	M-H	593.152	C_27_H_30_O_15_	Biorobin	RTATXGUCZHCSNG-UHFFFAOYSA-N	Bupleurum chinenseDC
27	2.0999	M-H	121.029	C_7_H_6_O_2_	Benzoic acid	WPYMKLBDIGXBTP-UHFFFAOYSA-N	Paeonia lactiflora Pall
28	2.1036	M + H	317.065	C_16_H_12_O_7_	Isorhamnetin	IZQSVPBOUDKVDZ-UHFFFAOYSA-N	Bupleurum chinenseDC, *Chrysanthemum* morifolium Ramat, *Glycyrrhiza* uralensis Fisch
29	2.1743	M-H	577.157	C_27_H_30_O_14_	Isorhoifolin	FKIYLTVJPDLUDL-UHFFFAOYSA-N	*Chrysanthemum* morifolium Ramat
30	2.1836	M + H	177.055	C_10_H_8_O_3_	1,4,5-Naphthalenetriol	NHEVNUARLCWEED-UHFFFAOYSA-N	*Glycyrrhiza* uralensis Fisch
31	2.3497	M-H	151.04	C_8_H_8_O_3_	2′,4′-Dihydroxyacetophenone	SULYEHHGGXARJS-UHFFFAOYSA-N	Angelica sinensis (Oliv.) Diels, Paeonia suffruticosa Andr
32	2.3527	M + H	153.054	C_8_H_8_O_3_	3′,4′-Dihydroxyacetophenone	UCQUAMAQHHEXGD-UHFFFAOYSA-N	*Chrysanthemum* morifolium Ramat
33	2.3766	M + H	447.092	C_21_H_18_O_11_	Flavone base + 3O, O-HexA	IKIIZLYTISPENI-UHFFFAOYSA-N	Bupleurum chinenseDC
34	2.5692	M-H	187.097	C_9_H_16_O_4_	Azelaic acid	BDJRBEYXGGNYIS-UHFFFAOYSA-N	Angelica sinensis (Oliv.) Diels
35	2.6237	M-H	137.024	C_7_H_6_O_3_	Salicylic acid	YGSDEFSMJLZEOE-UHFFFAOYSA-N	Paeonia lactiflora Pall
36	2.6862	M + H	133.101	C_10_H_14_O	Cuminyl alcohol	OIGWAXDAPKFNCQ-UHFFFAOYSA-N	*Chrysanthemum* morifolium Ramat
37	2.7094	M + H	338.138	C_20_H_20_NO_4+_	Jatrorrhizine	MXTLAHSTUOXGQF-UHFFFAOYSA-O	*Glycyrrhiza* uralensis Fisch
38	2.9293	M-H	255.066	C_15_H_12_O_4_	Pinocembrin	URFCJEUYXNAHFI-UHFFFAOYSA-N	Bupleurum chinenseDC, *Glycyrrhiza* uralensis Fisch
39	2.9846	M + H	165.091	C_10_H_12_O_2_	Eugenol	RRAFCDWBNXTKKO-UHFFFAOYSA-N	Bupleurum chinenseDC, Acorus tatarinowii Schott
40	3.0956	M + H	225.112	C_12_H_16_O_4_	Acoramone	AQZHZTTUVYQMIN-UHFFFAOYSA-N	Acorus tatarinowii Schott
41	3.1111	M-H	285.077	C_16_H_14_O_5_	Licochalcone B	DRDRYGIIYOPBBZ-XBXARRHUSA-N	*Glycyrrhiza* uralensis Fisch
42	3.1251	M-H	285.04	C_15_H_10_O_6_	Kaempferol	IYRMWMYZSQPJKC-UHFFFAOYSA-N	Bupleurum chinenseDC, Angelica sinensis (Oliv.) Diels, *Glycyrrhiza* uralensis Fisch, Paeonia lactiflora Pall, *Chrysanthemum* morifolium Ramat, Paeonia suffruticosa Andr
43	3.1532	M + H	431.133	C_22_H_22_O_9_	Formononetin-7-O-glucoside	MGJLSBDCWOSMHL-UHFFFAOYSA-N	*Glycyrrhiza* uralensis Fisch
44	3.1722	M + H	257.081	C_15_H_12_O_4_	Isoliquiritigenin	DXDRHHKMWQZJHT-FPYGCLRLSA-N	*Glycyrrhiza* uralensis Fisch
45	3.4099	M + H	177.054	C_10_H_8_O_3_	7-Methoxycoumarin	LIIALPBMIOVAHH-UHFFFAOYSA-N	Bupleurum chinenseDC
46	3.5486	M-H	201.113	C_10_H_18_O_4_	Sebacic acid	CXMXRPHRNRROMY-UHFFFAOYSA-N	Angelica sinensis (Oliv.) Diels
47	3.7276	M-H	221.081	C_12_H_14_O_4_	Diethyl-phthalate	FLKPEMZONWLCSK-UHFFFAOYSA-N	Bupleurum chinenseDC, *Chrysanthemum* morifolium Ramat, Paeonia suffruticosa Andr
48	3.8035	M + H	366.171	C_22_H_24_NO_4+_	Dehydrocorydaline	RFKQJTRWODZPHF-UHFFFAOYSA-N	*Chrysanthemum* morifolium Ramat
49	3.9343	M-H	823.412	C_42_H_64_O_16_	Zizyberanalic acid	SLWCVFLNZDOMEZ-UHFFFAOYSA-N	*Glycyrrhiza* uralensis Fisch
50	3.9481	M-H	283.06	C_16_H_12_O_5_	5,7-dihydroxy-6-methoxy-2-phenylchromen-4-one	LKOJGSWUMISDOF-UHFFFAOYSA-N	Bupleurum chinenseDC
51	3.9875	M + H	352.154	C_21_H_22_NO_4_	Palmatine	QUCQEUCGKKTEBI-UHFFFAOYSA-N	*Glycyrrhiza* uralensis Fisch
52	4.0611	M + H	273.076	C_15_H_12_O_5_	Naringenin	FTVWIRXFELQLPI-UHFFFAOYSA-N	Poria cocos (Schw.)Wol£, *Chrysanthemum* morifolium Ramat
53	4.2303	M + H	237.075	C_12_H_12_O_5_	6,7,8-trimethoxychromen-2-one	RAYQKHLZHPFYEJ-UHFFFAOYSA-N	Bupleurum chinenseDC
54	4.3339	M-H	821.394	C_42_H_62_O_16_	Licoricesaponin H2	LPLVUJXQOOQHMX-UHFFFAOYSA-N	*Glycyrrhiza* uralensis Fisch
55	4.3684	M-H	341.108	C_12_H_22_O_11_	Sucrose	CZMRCDWAGMRECN-UHFFFAOYSA-N	Paeonia lactiflora Pall
56	4.3808	M-H	515.119	C_25_H_24_O_12_	Dicaffeoyl quinic acid	YDDUMTOHNYZQPO-UHFFFAOYSA-N	*Chrysanthemum* morifolium Ramat
57	4.3913	M-H	283.06	C_16_H_12_O_5_	Genkwanin	JPMYFOBNRRGFNO-UHFFFAOYSA-N	*Glycyrrhiza* uralensis Fisch
58	4.4182	M + H	419.096	C_20_H_18_O_10_	Kaempferol 3-alpha-L-arabinofuranoside	POQICXMTUPVZMX-UHFFFAOYSA-N	Bupleurum chinenseDC
59	4.5988	M + H	167.07	C_9_H_10_O_3_	Paeonol	UILPJVPSNHJFIK-UHFFFAOYSA-N	Paeonia lactiflora Pall, Paeonia suffruticosa Andr
60	4.9284	M-H	837.389	C_42_H_62_O_17_	Licoricesaponin G2	WBQVRPYEEYUEBQ-UHFFFAOYSA-N	*Glycyrrhiza* uralensis Fisch
61	5.4779	M + H	927.532	C_48_H_78_O_17_	2-[4,5-Dihydroxy-6-[[8-hydroxy-8a-(hydroxymethyl)-4,4,6a,6b,11,11,14b-heptamethyl-1,2,3,4a,5,6,7,8,9,10,12,14a-dodecahydropicen-3-yl]oxy]-2-[[3,4,5-trihydroxy-6-(hydroxymethyl)oxan-2-yl]oxymethyl]oxan-3-yl]oxy-6-methyloxane-3,4,5-triol	PYJMYPPFWASOJX-UHFFFAOYSA-N	*Glycyrrhiza* uralensis Fisch
62	5.5239	M + H	487.342	C_30_H_46_O_5_	24-Hydroxyglycyrrhetic acid	GSEPOEIKWTXTHS-UHFFFAOYSA-N	Bupleurum chinenseDC
63	5.5502	M-H	807.416	C_42_H_64_O_15_	Oleanane -2H, +1O, 1COOH, O-HexA-HexA	BCNKILSUUHWRTG-UHFFFAOYSA-N	*Glycyrrhiza* uralensis Fisch
64	5.5703	M-H	247.134	C_15_H_20_O_3_	Atractylenolide III	FBMORZZOJSDNRQ-UHFFFAOYSA-N	*Glycyrrhiza* uralensis Fisch
65	5.6205	M-H	343.082	C_18_H_16_O_7_	Eupatilin	DRRWBCNQOKKKOL-UHFFFAOYSA-N	Poria cocos (Schw.)Wol£, Atractylodes macrocephala Koidz
66	5.8666	M + H	221.08	C_12_H_12_O_4_	Eugenitin	RGTSAUBIQAKKLC-UHFFFAOYSA-N	*Chrysanthemum* morifolium Ramat
67	6.0101	M-H	283.06	C_16_H_12_O_5_	Wogonin	XLTFNNCXVBYBSX-UHFFFAOYSA-N	Paeonia lactiflora Pall
68	6.2092	M + H	181.086	C_10_H_12_O_3_	Ethyl p-anisate	FHUODBDRWMIBQP-UHFFFAOYSA-N	Bupleurum chinenseDC
69	6.2988	M + H	285.075	C_16_H_12_O_5_	Glycitein	DXYUAIFZCFRPTH-UHFFFAOYSA-N	Lycium barbarum L
70	6.5724	M + H	355.127	C_20_H_18_O_6_	Isolicoflavonol	PGCKDCPTJAQQSQ-UHFFFAOYSA-N	Lycium barbarum L
71	6.6098	M-H	779.455	C_42_H_68_O_13_	Saikosaponin b2	WRYJYFCCMSVEPQ-UHFFFAOYSA-N	*Glycyrrhiza* uralensis Fisch
72	6.6421	M + H	121.064	C_8_H_8_O	Phenylacetaldehyde	DTUQWGWMVIHBKE-UHFFFAOYSA-N	Bupleurum chinenseDC
73	6.6678	M + H	369.132	C_21_H_20_O_6_	Isoglycycoumarin	PHHAXWBLJNBVNS-UHFFFAOYSA-N	Bupleurum chinenseDC
74	7.1121	M + H	353.138	C_21_H_20_O_5_	Glyurallin A	AEAIWNGAMDGGNB-UHFFFAOYSA-N	*Glycyrrhiza* uralensis Fisch
75	7.1387	M + H	193.085	C_11_H_12_O_3_	Myristicin	BNWJOHGLIBDBOB-UHFFFAOYSA-N	*Glycyrrhiza* uralensis Fisch
76	7.1421	M + H	389.123	C_20_H_20_O_8_	Artemetin	RIGYMJVFEJNCKD-UHFFFAOYSA-N	Angelica sinensis (Oliv.) Diels, *Chrysanthemum* morifolium Ramat
77	7.3535	M + H	339.159	C_21_H_22_O_4_	Licochalcone C	WBDNTJSRHDSPSR-KPKJPENVSA-N	*Chrysanthemum* morifolium Ramat
78	7.4898	M + H	367.117	C_21_H_18_O_6_	Glycyrol	LWESBHWAOZORCQ-UHFFFAOYSA-N	*Glycyrrhiza* uralensis Fisch
79	7.9405	M + H	279.232	C_18_H_30_O_2_	Alpha-Linolenic acid	DTOSIQBPPRVQHS-PDBXOOCHSA-N	*Glycyrrhiza* uralensis Fisch
80	8.5326	M + H	337.107	C_20_H_16_O_5_	Glabrone	COLMVFWKLOZOOP-UHFFFAOYSA-N	Bupleurum chinenseDC, Lycium barbarum L
81	8.7257	M-H	315.253	C_17_H_34_O_2_	Methyl hexadecanoate	FLIACVVOZYBSBS-UHFFFAOYSA-N	*Glycyrrhiza* uralensis Fisch
82	8.788	M + H	381.206	C_24_H_28_O_4_	Levistilide A	UBBRXVRQZJSDAK-DZWUWFSDSA-N	*Chrysanthemum* morifolium Ramat, Lycium barbarum L
83	9.5661	M-H	419.15	C_25_H_24_O_6_	Morusin	XFFOMNJIDRDDLQ-UHFFFAOYSA-N	Angelica sinensis (Oliv.) Diels
84	10.0958	M + H	149.023	C8H4O3	Phthalic anhydride	LGRFSURHDFAFJT-UHFFFAOYSA-N	*Glycyrrhiza* uralensis Fisch
85	10.6272	M + H	191.106	C_12_H_14_O_2_	3-n-Butylphathlide	HJXMNVQARNZTEE-UHFFFAOYSA-N	Angelica sinensis (Oliv.) Diels
86	10.631	M + H	307.263	C_20_H_34_O_2_	Linolenic acid ethyl ester	JYYFMIOPGOFNPK-UHFFFAOYSA-N	Angelica sinensis (Oliv.) Diels
87	11.609	M + H	121.065	C_8_H_8_O	Acetophenone	KWOLFJPFCHCOCG-UHFFFAOYSA-N	Lycium barbarum L
88	11.8328	M-H	455.353	C_30_H_48_O_3_	Oleanolic acid	MIJYXULNPSFWEK-UHFFFAOYSA-N	Bupleurum chinenseDC, Angelica sinensis (Oliv.) Diels
89	12.3894	M + H	135.116	C_10_H_14_	p-Cymene	HFPZCAJZSCWRBC-UHFFFAOYSA-N	Bupleurum chinenseDC, Angelica sinensis (Oliv.) Diels, Paeonia lactiflora Pall, Paeonia suffruticosa Andr
90	12.6579	M-H	469.331	C_30_H_46_O_4_	Enoxolone	MPDGHEJMBKOTSU-UHFFFAOYSA-N	Bupleurum chinenseDC, Angelica sinensis (Oliv.) Diels, *Chrysanthemum* morifolium Ramat
91	13.5409	M + H	240.232	C_15_H_26_O	Beta-Caryophyllene Alcohol	FUQAYSQLAOJBBC-UHFFFAOYSA-N	*Glycyrrhiza* uralensis Fisch
92	15.1919	M + H	118.086	C_5_H_11_NO_2_	Valine	KZSNJWFQEVHDMF-UHFFFAOYSA-N	*Chrysanthemum* morifolium Ramat
93	15.2072	M + H	127.039	C_6_H_6_O_3_	5-Hydroxymethylfurfural	NOEGNKMFWQHSLB-UHFFFAOYSA-N	Atractylodes macrocephala Koidz

### Establishment of CUMS rat model

**FIGURE 3:**
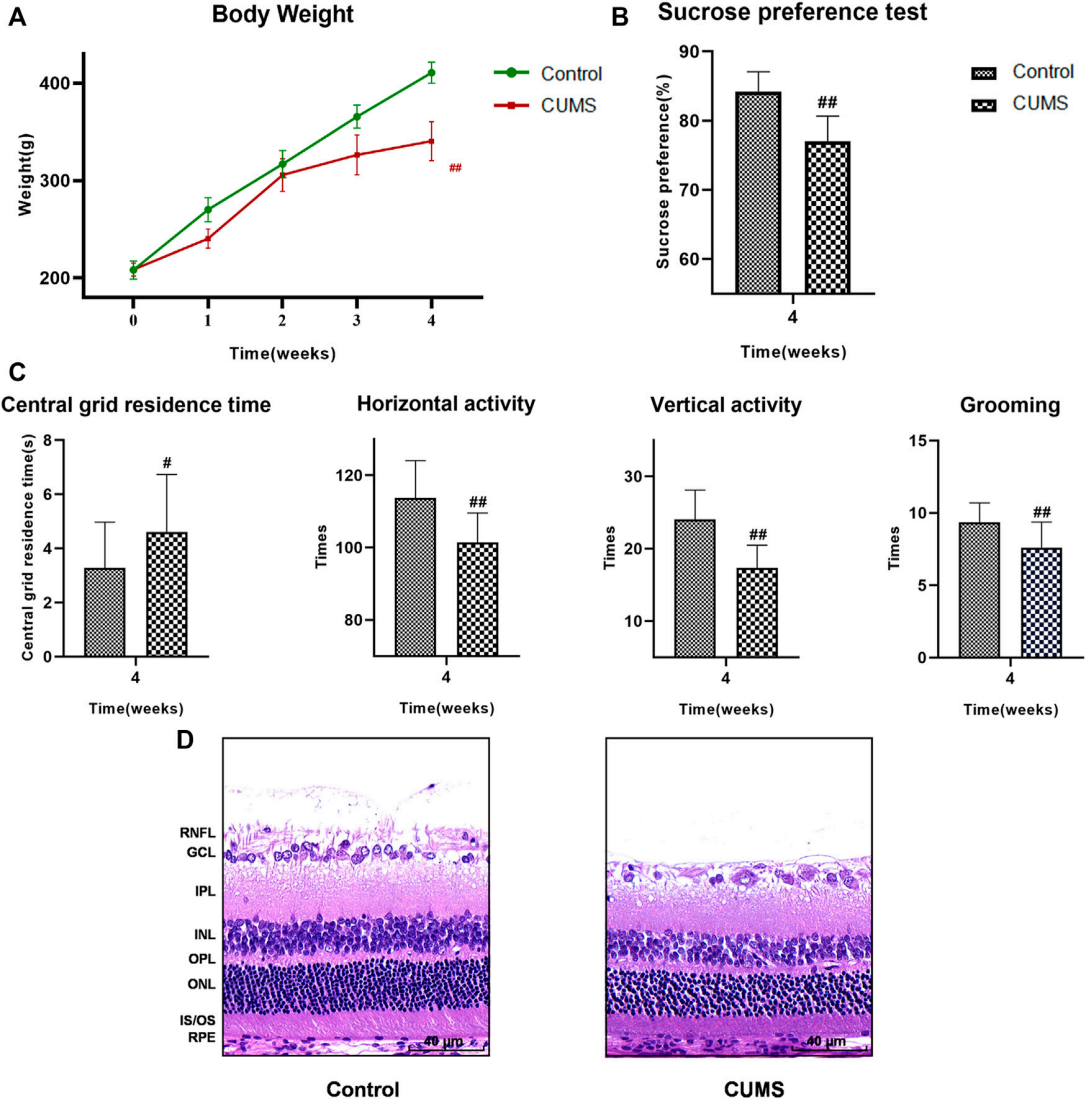
At 4 weeks, the results of weight, SPT, OFT and HE staining of the rats in the control group and CUMS group. **(A)** Weight trend of rats in the two groups. **(B)** Results of SPT. **(C)** Results of OFT, including the central grid residence time, horizontal activity, vertical activity and grooming times. (Control group n = 14, CUMS group n = 38). Results are shown as the mean ± SD. ^**#**^ *p* < 0.05 and ^**##**^ *p* < 0.01 vs. control group. **(D)** HE staining of retina in the two groups. Scale bar = 40 µm. In the CUMS group, the full retina was thinner; the nucleus of RGC was pyknotic and the number of RGC decreased; the INL and ONL were loose and irregular. RNFL, retinal nerve fiber layer; GCL, ganglion cell layer; RGC, retinal ganglion cell; IPL, inner plexiform layer; INL, inner nuclear layer; OPL, outer plexiform layer; ONL, outer nuclear layer; IS/OS, the inner and outer segments of photoreceptors; RPE, retinal pigmented epithelium.

Four weeks into the experiment, all rats underwent weight measurement, SPT and OFT. Body weight and sucrose preference were lower in the CUMS group than the control group (Figures 3A,B, *p* < 0.01). Results of the OFT showed that the central grid residence time was significantly longer, while the horizontal and vertical activity and grooming times were significantly reduced in the CUMS group compared with controls (Figure 3C, *p* < 0.05) indicating that the CUMS model was successful.

The results of HE staining showed that the whole retina of CUMS group was thinner than that of normal group. Nuclear pyknosis of the retinal ganglion cell (RGC) was seen, the number and volume of RGC were decreased, and the cytoplasm was reduced. The inner plexiform layer (IPL) was thinned, while INL and ONL were loose and irregular. The results suggested that the retinas of CUMS rats were thin and morphologically abnormal (Figure 3D).

### Effects of MMXY on body weight of CUMS rats

**FIGURE 4:**
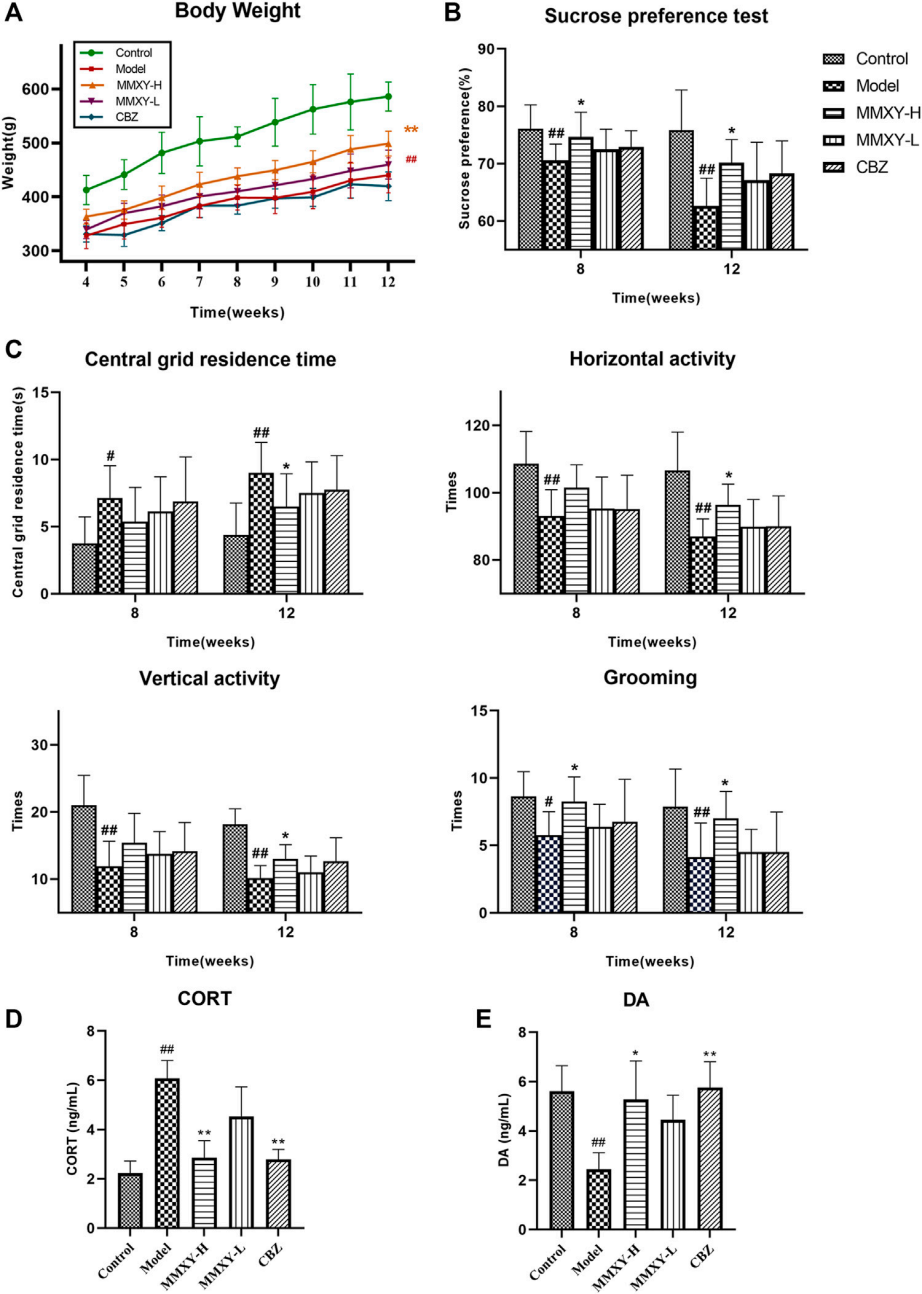
At 4–12 weeks, effects of MMXY on body weight, SPT, OFT, CORT and DA of CUMS rats in control group, model group, MMXY-H group, MMXY-L and CBZ groups. **(A)** Weight of rats at 4-12 weeks (n = 8). **(B)** SPT results at weeks 8 and 12 (n = 8). **(C)** OFT results at weeks 8 and 12, including the central grid residence time, horizontal activity, vertical activity and grooming times (n = 8). Results are shown as the mean ± SD. ^**#**^ *p* < 0.05 and ^**##**^ *p* < 0.01 vs. control group; *p < 0.05 and **p < 0.01 vs. model group. **(D,E)** At 12 weeks, the level of CORT and DA in each group (n = 8). Results are shown as the median (P25, P75). ^**#**^ *p* < 0.05 and ^**##**^ *p* < 0.01 vs. control group; *p < 0.05 and **p < 0.01 vs. model group.

At week 4, there was no significant difference in body weight between the CUMS groups. However, with the persistence of stimulation and intervention, body weight began to differ between the groups. At week 12, the bodyweight of the model group was significantly lower than that of the control group (*p* < 0.01), while the body weight of the MMXY-H group was significantly higher than that of the model group (*p* < 0.01). Figure 4A showed the changes in bodyweight in each group during weeks 4–12.

### Effects of MMXY on sucrose preference in CUMS rats

The SPT was used to assess the rat’s reactivity to reward. At weeks 8 and 12, the sucrose preference of the model group was significantly lower than that of the control group (*p* < 0.01), whereas MMXY-H reversed the sucrose preference of rats compared with the model group (*p* < 0.05). (Figure 4B).

### Effects of MMXY on open field activity of CUMS rats

At weeks 8 and 12, the central grid residence time of the model group was significantly longer than that of the control group, and the horizontal activity, vertical activity and grooming times in the model group were significantly lower than those in the control group (*p* < 0.05). At week 12, MMXY-H had significantly improved the open field activity of rats in all of the above aspects compared with the model group (*p* < 0.05). (Figure 4C).

### Effects of MMXY on serum CORT and DA levels in CUMS rats

At week 12, the serum CORT level of the model group was significantly higher than that of the control group (*p* < 0.01), whereas MMXY and CBZ significantly reduced serum CORT level than that of the model group. (Figure 4D, *p* < 0.01). Moreover, the serum DA level of the model group was significantly lower than that of the control group (*p* < 0.01), indicating that the rats’ excitability was significantly decreased, whereas MMXY-H and CBZ significantly increased the serum DA level of CUMS rats compared with the model group (Figure 4E, *p* < 0.05).

### Effects of MMXY on tissue morphology and retinal thickness in CUMS rats

**FIGURE 5:**
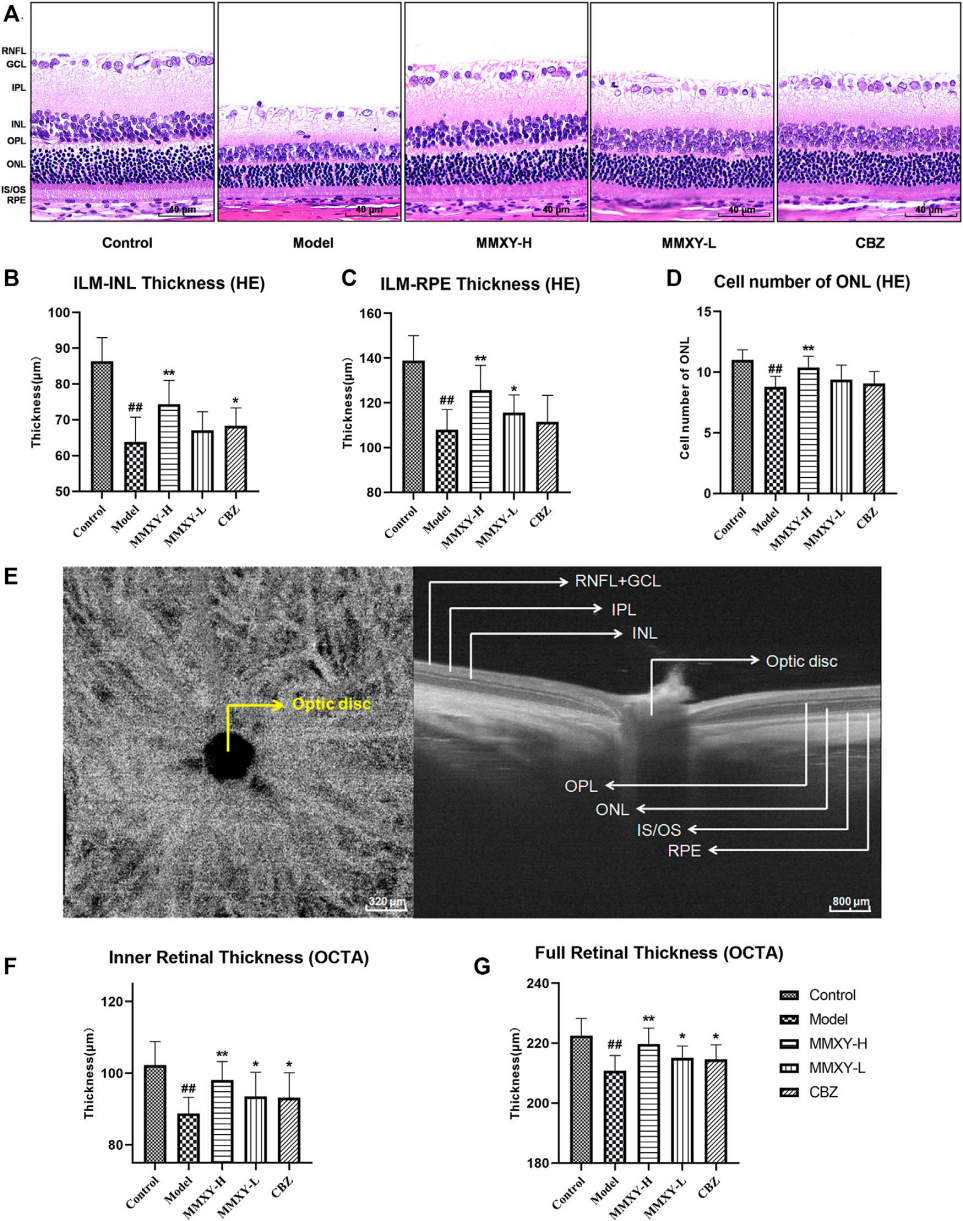
Effects of MMXY on morphology and thickness of the retina in CUMS rats. **(A)** HE staining morphology and thickness of retina in each group. Scale bars = 40um. **(B,C)** The thickness of ILM-INL and ILM-RPE in HE staining (n = 18 points). **(D)** HE staining showed the number of ONL cells in each group (n = 18 points). Results are shown as the mean ± SD. ^**#**^ *p* < 0.05 and ^**##**^ *p* < 0.01 vs. control group; *p < 0.05 and **p < 0.01 vs. model group. **(E)** B-scan image of rat retina in OCTA. Scale bars = 320/800 µm. **(F,G)** OCTA results of the inner and full thickness of retina in each group (n = 16 eyes). Results are shown as the mean ± SD. ^**#**^ *p* < 0.05 and ^**##**^ *p* < 0.01 vs. control group; *p < 0.05 and **p < 0.01 vs. model group.

At week 12, under light microscopy the boundaries of retinal layers were clear in the control group. RGC cytoplasm was abundant, RGCs were uniformly arranged, and the nuclear membrane was clear. INL and ONL were evenly arranged, the nuclear membrane was smooth and complete, and the chromatin was uniform. Inner and outer plexiform layers and the inner and outer segments of photoreceptors (IS/OS) were uniformly stained. In the model group, the retina was noticeably thinner, RGCs showed nuclear pyknosis, small cell volume and little cytoplasm. The IPL was thinner, and cells of INL and ONL decreased in number and volume. By comparison, in the MMXY-H group the retinal morphology was significantly recovered, and comparable with that of the control group, while in the MMXY-L group the morphology and number of cells in INL and ONL were restored to normal. However, the number of RGC remained low, with vacuolar degeneration, pyknosis of some cell nuclei and shrunken or absent cell cytoplasm. In the CBZ group the ganglion cell layer (GCL) had largely returned to normal, but INL and ONL showed mild thinning (Figure 5A).

On HE stained sections, compared with the control group, the thickness of inner limiting membrane (ILM)-INL (the inner retina) and ILM-RPE (the full retina) were significantly decreased in the model group (*p* < 0.01), and the number of ONL was significantly reduced (*p* < 0.01), whereas MMXY-H recovered the thickness of each layer and the number of ONL compared with the model group (*p* < 0.01). (Figures 5B–D).

Figure 5E shows OCTA centered on the optic disc. Compared with the control group, thickness of inner and full retina were reduced in the model group, whereas MMXY restored the retinal thickness compared with the model group (*p* < 0.05). (Figures 5F,G).

### Effects of MMXY on blood flow of inner retina in CUMS rats

**FIGURE 6:**
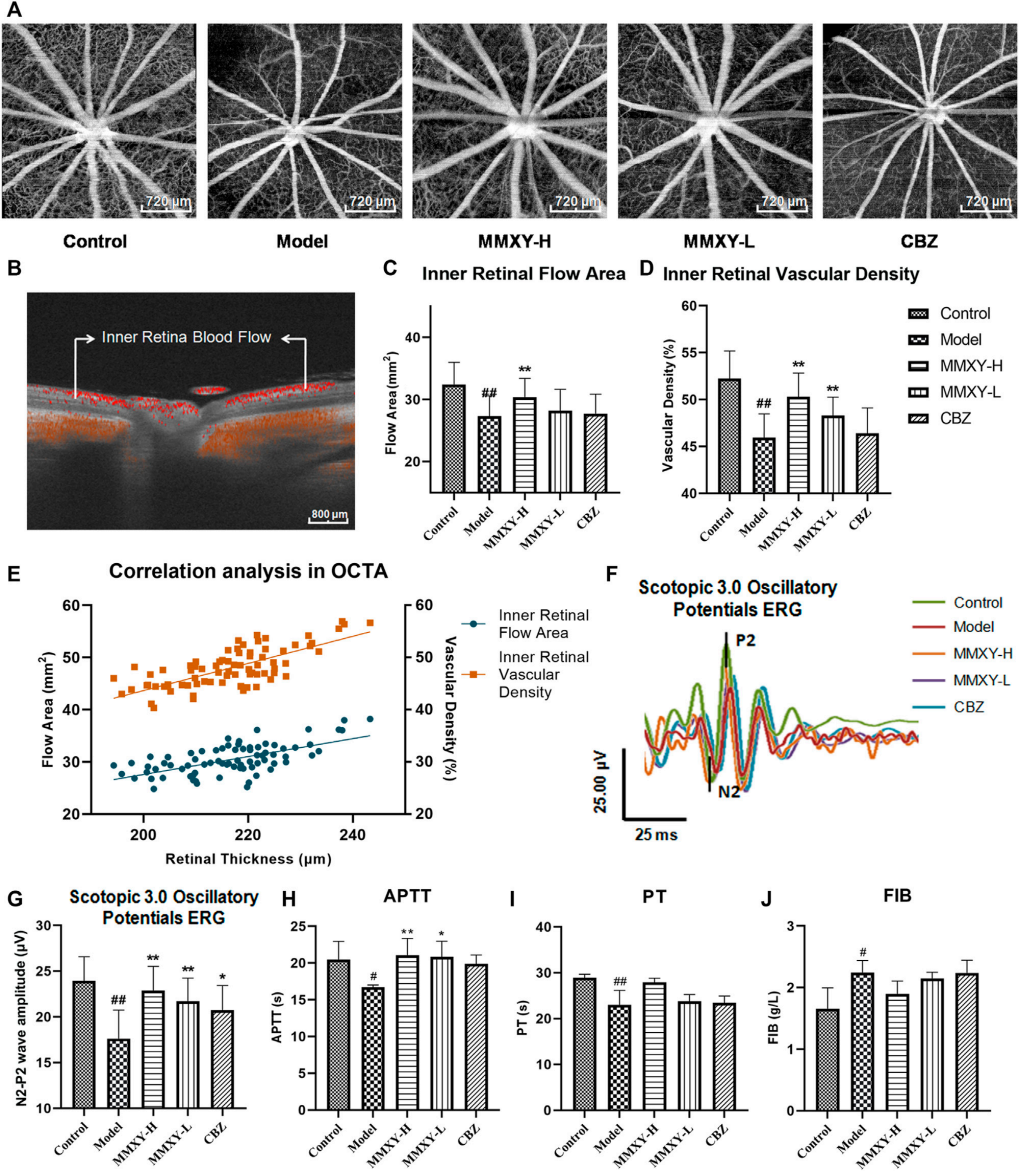
Effect of MMXY on retinal blood flow in CUMS rats. **(A)** Inner retina enhanced angio image in OCTA. Scale bars = 720 µm. **(B)** OCTA B-scan of retinal blood flow. Scale bars = 800 µm **(C,D)** The inner retinal blood flow area and vascular density in each group (n = 16 eyes). Results are shown as the mean ± SD. **(E)** Correlation analysis of inner retinal flow area and vascular density with retinal thickness (r_green_ = 0.63 *p* < 0.01, r_orange_ = 0.73 *p* < 0.01). **(F,G)** Effects of MMXY on OPs waveform curve and amplitude in CUMS rats (n = 8 eyes). Results are shown as the mean ± SD. ^**#**^ *p* < 0.05 and ^**##**^ *p* < 0.01 vs. control group; *p < 0.05 and **p < 0.01 vs. model group **(H–J)** Effects of MMXY on APTT, PT and FIB in CUMS rats (n = 6 rats). Results are shown as the median (P25, P75). ^**#**^ *p* < 0.05 and ^**##**^ *p* < 0.01 vs. control group; *p < 0.05 and **p < 0.01 vs. model group.

Figures 6A,B show inner retinal blood flow area and vascular density. Compared with the control group, the inner retinal blood flow area and vascular density of rats in the model group were significantly decreased (*p* < 0.01), and MMXY increased these values compared with the model group (*p* < 0.05) (Figures 6C,D). The flow area (green pots) and vascular density (orange pots) of inner retinal layer were positively correlated with retinal thickness (r_green_ = 0.63 *p* < 0.01, r _orange_ = 0.73 *p* < 0.01) (Figure 6E).

In Scotopic 3.0 oscillatory potentials ERG, the amplitude of the second wave of the oscillatory potentials (OPs2) is reduced when the inner retinal layer is ischemic (Nagai et al., 2017). The amplitude of the OPs2 (N2-P2) in the model group was significantly lower than that in the control group (*p* < 0.01). This suggests poor inner retinal blood supply in the model group. In the MMXY group, the amplitude of the OPs2 wave was increased compared with the model group (*p* < 0.01) (Figures 6F,G), which implied that MMXY could increase the inner retinal blood supply. ERG results were therefore consistent with OCTA results.

At week 12, compared with the control group, APTT and PT in the model group were significantly decreased (*p* < 0.05), while FIB was significantly increased (*p* < 0.05), suggesting that the model group was in a hypercoagulable state. However, MMXY can increase APTT compared with the model group (*p* < 0.05), indicating that the hypercoagulable state can be alleviated (Figures 6H–J).

### Effects of MMXY on ERG in CUMS rats

**FIGURE 7:**
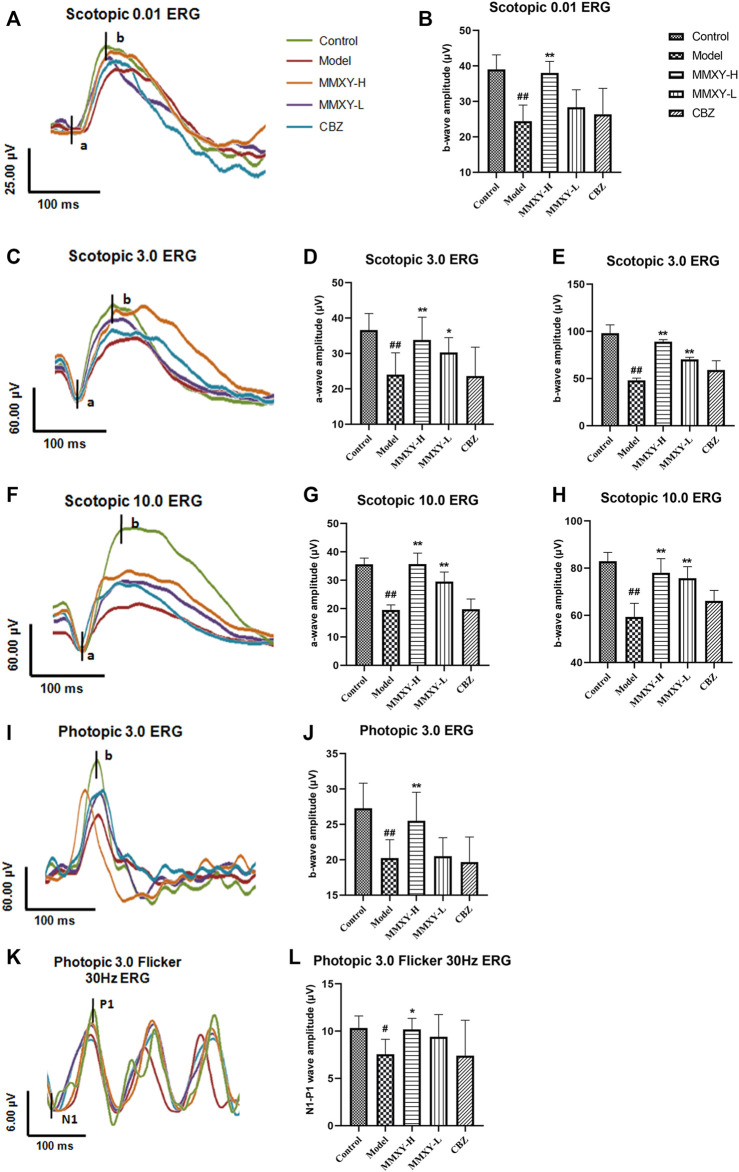
Effects of MMXY on ERG of CUMS rats in each group. **(A)** The waveform curve of Scotopic 0.01 ERG. **(B)** The b-wave amplitude of Scotopic 0.01 ERG (n = 8). **(C)** The waveform curve of Scotopic 3.0 ERG. **(D,E)** The a- and b-wave amplitudes of Scotopic3.0 ERG (n = 8). **(F)** The waveform curve of Scotopic 10.0 ERG. **(G,H)** The a- and b-wave amplitudes of Scotopic10.0 ERG (n = 8). **(I)** The waveform curve of Photopic 3.0 ERG. **(J)**The b-wave amplitude of Photopic 3.0 ERG (n = 8). **(K)** The waveform curve of Photopic 3.0 flicker 30 Hz ERG. **(L)** The N1-P1 wave amplitude of Photopic 3.0 flicker30 Hz ERG (n = 8). In the waveform curve, the length of the horizontal scale represents time (25-100 ms), and the length of the longitudinal scale represents amplitude (4-60 μV). Different colors represent different groups. Results are shown as the mean ± SD. ^**#**^ *p* < 0.05 and ^**##**^ *p* < 0.01 vs. control group; *p < 0.05 and **p < 0.01 vs. model group.

Scotopic ERG tests mainly reflect the function of rod cells, while photopic ERG tests mainly reflect the function of cone cells (Marmor et al., 2009). Compared with the control group, the amplitude of each wave (both scotopic and photopic) in the model group was significantly decreased (*p* < 0.05), suggesting impaired function of the retinal cone and rod cells. However, the amplitude of each wave in the MMXY group was increased compared with the model group (*p* < 0.05), which indicates that MMXY may improve the function of the cone and rod cells (Figure 7).

### Effects of MMXY on conduction function of visual pathway in CUMS rats

**FIGURE 8:**
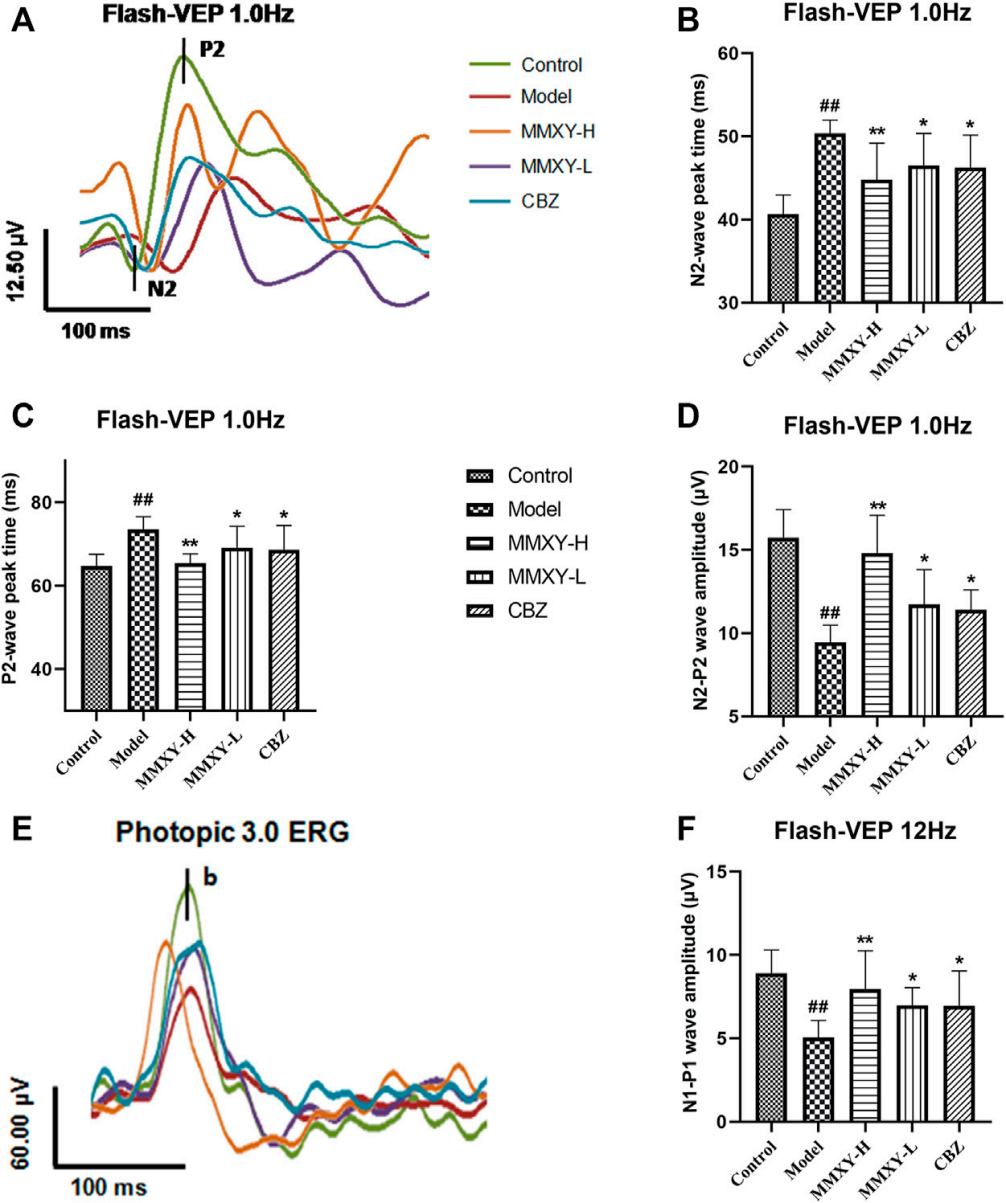
Effects of MMXY on VEP of CUMS rats in each group. **(A)** The waveform curve of Flash-VEP 1.0 Hz. **(B,C)** N2 and P2 Peak times of Flash-VEP 1.0 Hz (n = 8 eyes). **(D)** The N2-P2 wave amplitude of Flash-VEP 1.0 Hz (n = 8 eyes). **(E)** The waveform curve of Flash-VEP 12 Hz. **(F)** The N1-P1wave amplitude of Flash-VEP 12 Hz (n = 8 eyes). Results are shown as the mean ± SD. ^**#**^ *p* < 0.05 and ^**##**^ *p* < 0.01 vs. control group; *p < 0.05 and **p < 0.01 vs. model group.

VEPs reflect visual pathway function. Compared with the control group, the peak times of N2 and P2 in the model group were prolonged and the amplitude was decreased (*p* < 0.01), while peak times were reduced and amplitudes increased in the MMXY-H and CBZ groups compared with the model group (*p* < 0.05) (Figures 8A–D). After 12 Hz stimulation, amplitude changes showed the same trend (Figures 8E,F), indicating that MMXY and CBZ may improve visual pathway function in CUMS rats.

### Effects of MMXY on retinal ultrastructure in CUMS rats

**FIGURE 9:**
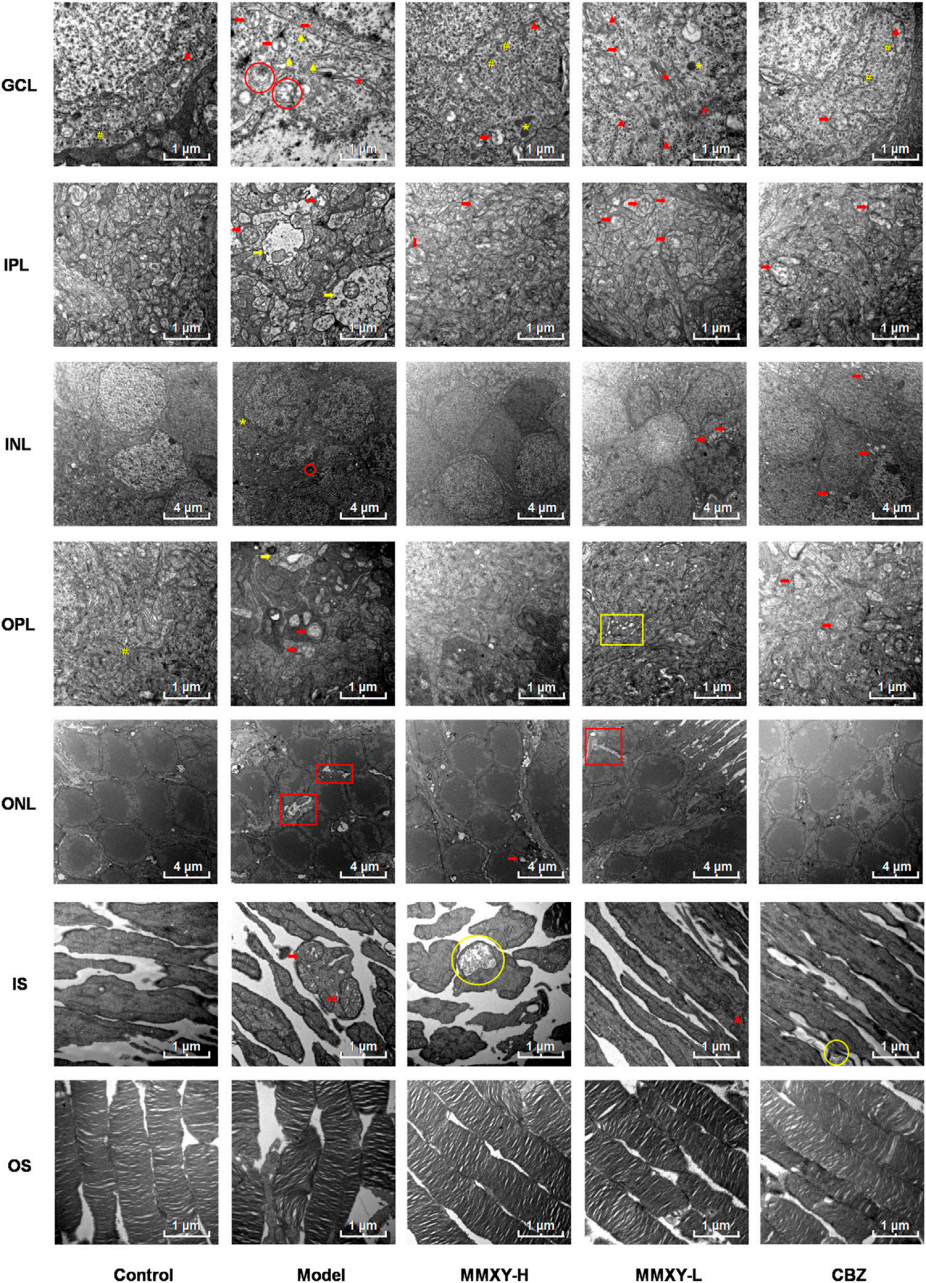
Electron microscope ultrastructure of rat retina. The ultrastructure of retina in model group was severely damaged. In the model group, the photoreceptors showed an increase in the spaces between their OSs and those between their membranous disks; Swollen mitochondrias (red arrow) and autophagosomes (red circle) were seen in GCL, and swollen synapses (yellow arrow) were seen in IPL and OPL. In MMXY-H group, the photoreceptors showed marked reduction in the spaces between their OSs and those between their membranous disks compared to model group, mitochondrial swelling was rare in GCL, and there was no obvious synaptic swelling in IPL and OPL. Red arrow, swollen mitochondria; Yellow arrow, Swollen neuron synapse; Red circle, autophagosomes; Yellow circle, glycogen granules; Red asterisk, Golgi; Yellow asterisk, lysosome; Yellow well, Poly ribosome; Red triangle, rough endoplasmic reticulum; Yellow triangle, smooth endoplasmic reticulum; Yellow box, endoplasmic reticulum swelling; Red box, interstitial swelling.

At 12 weeks, the retinal ultrastructure of rats in each group was as follows (Figure 9):

Control group: OS layer was composed of slender and regularly arranged membranous disks with minimal space between them. The IS layer was characterized by abundant mitochondria with normal structure. In ONL, the cell membrane was smooth, and the intercellular connections were tight. INL nuclear membrane was intact with a clear boundary and uniform euchromatin. No mitochondrial vacuolation was observed. Many poly ribosomes were found in the OPL. No neuronal swelling was observed in IPL. The GCL demonstrated electron dense euchromatin, rough endoplasmic reticulum, and poly ribosomes.

Model group: the space between OS and membranous disks was significantly increased; the mitochondria of IS layer were swollen and the cristae of mitochondria were broken. The intercellular stroma of ONL was swollen and loose, and tight junctions were reduced. There was INL cell membrane shrinkage, nuclear membrane invagination, nuclear pyknosis and significantly increased, heterochromatin. Autophagosomes were seen in the cytoplasm. In OPL and IPL, we observed synaptic swelling, mitochondrial swelling and vacuolated mitochondria, and reduced or even absent content. In GCL, mitochondria were swollen and vacuolated, smooth endoplasmic reticulum was dilated, the flattened sac of the Golgi apparatus was dilated, and autophagosomes were seen.

MMXY-H group: the space between OS and their membranous disks decreased significantly, glycogen particles were accumulated in the mitochondria, but mitochondrial swelling was observed only occasionally. The ONL cells were closely connected, also occasional mitochondrial swelling. In INL, the membrane was smooth, and the nuclear membrane was intact. No neuronal swelling was observed in OPL and IPL, and mitochondrial swelling was rare. The appearance of RGC was normal. In GCL, no mitochondrial swelling was apparent, rough endoplasmic reticulum was less degranulated, but a large number of poly ribosomes could be seen.

MMXY-L group: The space between OS and their membranous disks was slightly increased; in the IS layer, mitochondrial swelling was not apparent, and rough endoplasmic reticulum could be seen. In ONL, there was nuclear pyknosis and loose intercellular connections. In INL, the cell membrane was shrunken, and mild mitochondrial swelling could be seen within the nuclear membrane. No obvious neuronal swelling was observed in OPL and IPL, but mitochondria swelling, vacuolation and endoplasmic reticulum expansion were observed. In GCL, Golgi apparatus, lysosomes, large quantities of rough endoplasmic reticulum degranulation, mitochondrial swelling and vacuoles were observed.

CBZ group: the space between OS and their membranous disks was reduced. In IS layer, glycogen particle accumulation could be seen in the mitochondria. In ONL, the cone and rod cell nuclei could be observed. The cone nucleus, with low electron density, contained clumps of chromatin, while the rod nucleus, with high electron density, contained heterochromatin (Eltony and Abdelhameed, 2017). In INL, the mitochondria were swollen and vacuolated, but nuclear membrane depression was improved. Mitochondrial swelling was seen in OPL and IPL. In GCL, coarse endoplasmic reticulum degranulation and mitochondrial swelling were seen, and polyribosomes were observed.

The above results suggest that the retinal ultrastructure of the model group was significantly damaged, whereas MMXY restored its morphology.

### Effects of MMXY on retinal autophagy in CUMS rats

**FIGURE 10:**
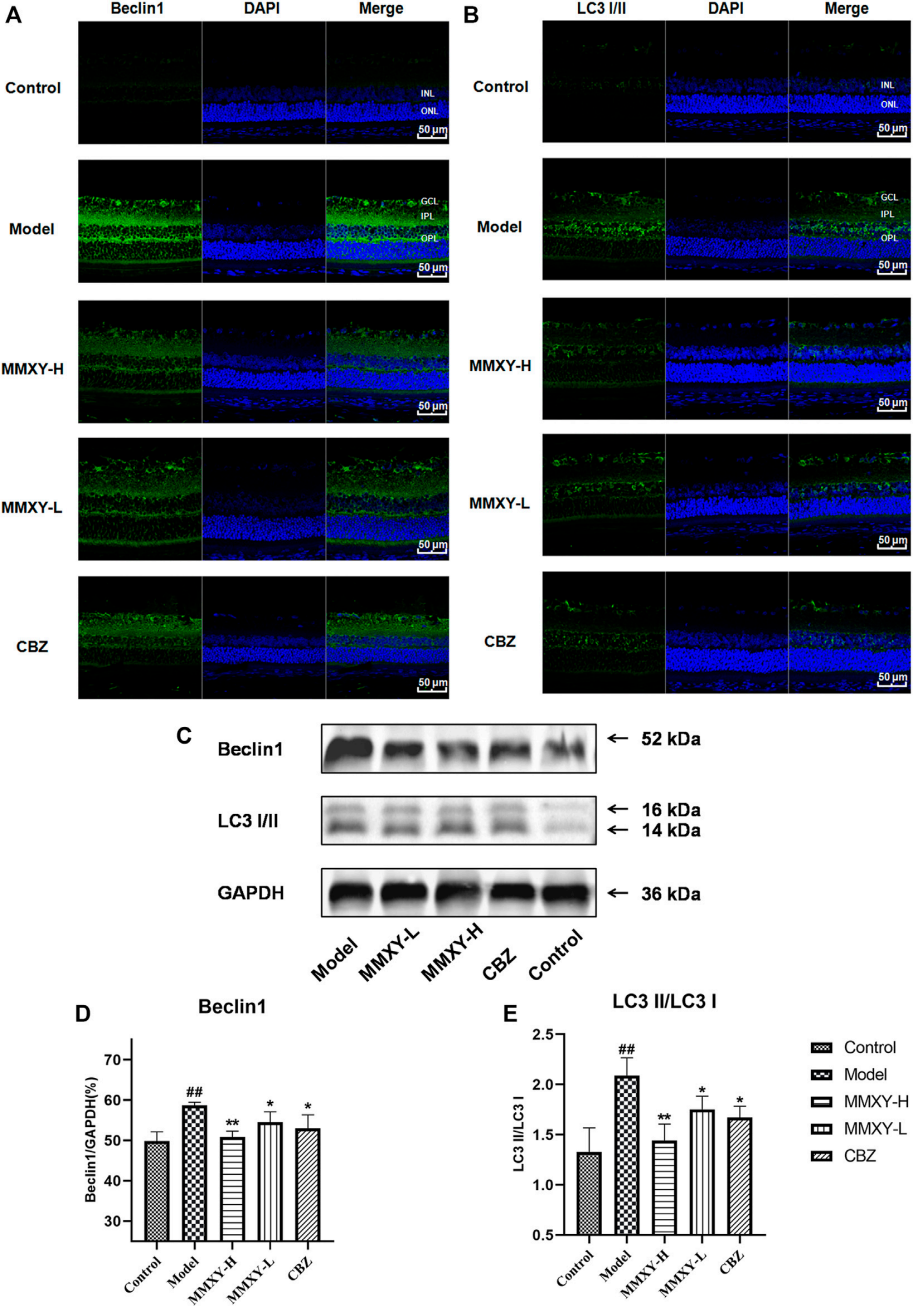
Effects of MMXY on retinal autophagy flux in CUMS rats **(A,B)** Immunofluorescence staining of Beclin1 (green), LC3 (green) and DAPI (blue) of retinas in each group. Scale bars = 50 µm. **(C)**Representative images of Western blot showing Beclin1 and LC3I/II expression in the retina of each group. GAPDH was used to confirm an equal amount of protein (n = 3). **(D,E)** Quantitative expression of Beclin1 and LC3II/LC3I in each group. Results are shown as the mean ± SD (n = 3). ^**#**^ *p* < 0.05 and ^**##**^ *p* < 0.01 vs. control group; *p < 0.05 and **p < 0.01 vs. model group.

In the ultrastructure of rat retinal electron microscopy, autophagosomes were easily found in GCL and INL of the model group (red circles in Figure 9), but were less apparent in other groups. The expression of Beclin1 and LC3 protein in the retina was detected by immunofluorescence. In the model group, compared with controls, the fluorescence intensity of Beclin1 protein in GCL, IPL, INL and OPL was significantly increased, and the fluorescence particles were apparent, whereas the fluorescence intensity was weak in the control group. The fluorescence of LC3 protein in the model group was significantly enhanced in GCL and INL, and the fluorescence particles were bright compared with the control group, which indicated that autophagy was enhanced in the model group. In the MMXY group, the fluorescence expression of Beclin1 and LC3 was significantly decreased, and the fluorescence particles were reduced (Figures 10A,B), suggesting that MMXY inhibited the expression of retinal autophagy.

Consistent with this, western blot results showed that Beclin1 and LC3-II/LC3I protein expressions were significantly increased in the model group (*p* < 0.01), while Beclin1 and LC3-II/LC3I protein expressions were significantly decreased in the MMXY group compared with the model group (*p* < 0.05) (Figures 10C–E), indicating that MMXY reduced autophagy.

### Effects of MMXY on PI3K-Akt-mTOR pathway in the retina of CUMS rats

**FIGURE 11:**
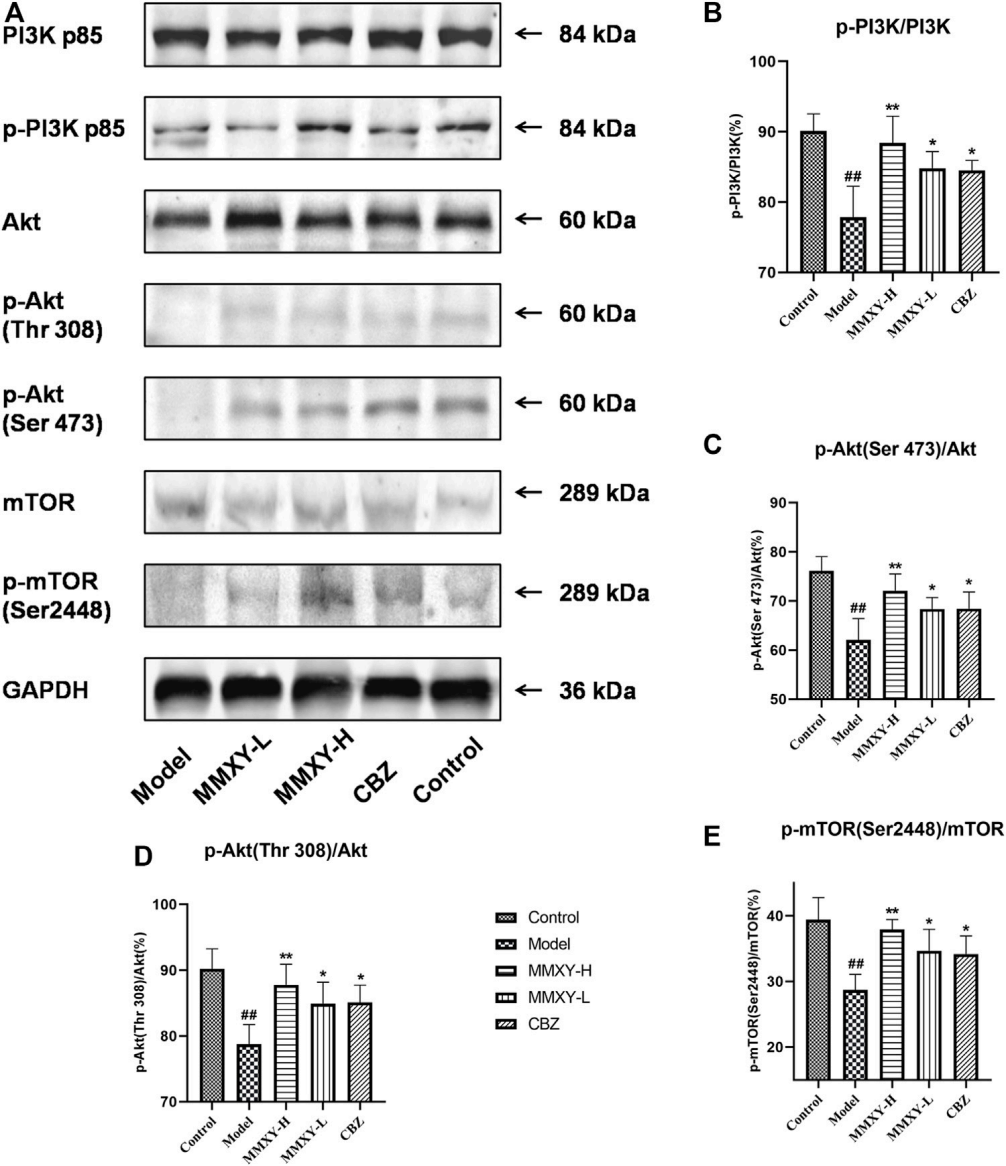
Effects of MMXY on protein expression of PI3K/Akt/mTOR pathway in retina of CUMS rats. **(A)** Representative images of Western blot showing PI3K p85, p-PI3K p85, Akt, p-Akt (Thr308), p-Akt (Ser473), mTOR, p-mTOR (Ser2448) expression in the retina of each group. GAPDH was used to confirm an equal amount of protein (n = 3). **(B–E)** Quantitative expression of p-PI3K/PI3K, p-Akt (Ser473)/Akt, p-Akt (Thr308)/Akt, p-mTOR (Ser2448)/mTOR in each group. ^**#**^ *p* < 0.05 and ^**##**^ *p* < 0.01 vs. control group; *p < 0.05 and **p < 0.01 vs. model group.

Figure 11A showed the phosphorylated protein and total protein of PI3K/Akt/mTOR in the retina. Compared with controls, the proportion of phosphorylated proteins in the model group decreased (*p* < 0.01), while the proportion of phosphorylated proteins in MMXY and CBZ increased compared with the model group (*p* < 0.05), suggesting that MMXY activated the PI3K-Akt-mTOR signaling pathway (Figures 11B–E).

## Discussion

The close association between many retinal diseases and psychosomatic diseases may be related to the patients’ reduced visual function (Moschos et al., 2015; Vu et al., 2021), self-assessment of health and quality of life due to the condition (Vu et al., 2021), and dependence on family caregivers (Jin et al., 2021). In addition, high mental stress (Xu et al., 2022), low income and living alone (Chen et al., 2018) are all risk factors for eye disease associated with psychosomatic disease, but increased social support has a significant protective effect on such patients (Xu et al., 2022). Negative emotions such as anxiety and depression can further exacerbate the progression of eye disease and anxiety reportedly increases the risk of glaucoma progression (Shin et al., 2021). Similarly, a significant association exists between depression and the risk of RVO progression, with a higher incidence of RVO among patients with recurrent depression (Ha et al., 2021). Progression of DR is significantly increased in diabetic patients with depression or depressive symptoms (Khoo et al., 2019). Conversely, the retinal nerve fiber layer (RNFL) and macula are thinner in major depressive disorder than in age - and sex-matched healthy subjects, and RNFL and macula thickness correlates with the severity of depression (Liu et al., 2022). These findings indicate that anxiety, depression and other negative emotions may be important risk factors for onset and progression of retinal diseases. The present study investigated the mechanisms and pathways linking negative emotions and exacerbation of retinal disease.

MMXY is made by adding and subtracting Xiaoyao Powder, which is a classic prescription with functions of soothing the liver, nourishing the blood and invigorating the spleen. Xiaoyao Powder is widely used in clinical treatment of anxiety and depression-like conditions. Animal studies show that the drug is effective in improving behavior in mice with symptoms of anxiety and depression (Liu et al., 2019; Liu et al., 2021). MMXY has the functions of soothing the liver and relieving depression, clearing the liver and brightening the eyes. It is composed of Xiaoyao Powder with Zingiber officinale Rosc. and Mentha haplocalyx Briq. removed, while adding *Chrysanthemum* morifolium Ramat., Lycium barbarum L., Paeonia suffruticosa Andr., and Acorus tatarinowii Schott. *Chrysanthemum* morifolium Ramat. and Lycium barbarum L. are both effective in the treatment of eye diseases. It has been reported that diosmetin in *Chrysanthemum* morifolium Ramat. protects against retinal damage (Shen et al., 2016). Lycium barbarum L. extract can inhibit oxidative stress, thereby improving retinal morphology and function (Yeh et al., 2020). Lycium barbarum L. has therapeutic effects on retinal neurodegeneration in rats with glaucoma or ocular hypertension (Chan et al., 2007). Paeonol, one of the main components of Paeonia suffruticosa Andr., has a protective effect on the myocardium by inhibiting cell apoptotic and autophagic cell death in a myocardial ischemia/reperfusion injury model (Tsai et al., 2020). In addition, paeonol not only has anti-anxiety function (Mi et al., 2005) but it also inhibits platelet aggregation and anticoagulation (Koo et al., 2010). Acorus tatarinowii Schott has apparent anti-myocardial ischemia function (Kang et al., 2006). Eugenol, an active component of Acorus tatarinowii Schott, shows antidepressant activity in mice (Tao et al., 2005). β-asarone, another active component of Acorus tatarinowii Schott, improves cognitive function in mice (Chen et al., 2014). Our study confirmed that MMXY not only effectively improves anxiety and depression-like behavior but also effectively protects retinal morphology and improves retinal function in CUMS rats. Carbamazepine is a commonly used anticonvulsant, but a double-blind study of 35 depressed patients showed that carbamazepine has some acute antidepressant efficacy and acute antimanic and longer-term prophylactic efficacy in both phases of manic-depressive illness (Post et al., 1986). Carbamazepine has a comparative advantage in patients with bipolar disorder and prevents new depressive or manic episodes (Grunze et al., 2021). Recent animal experiments have shown that Carbamazepine exerts an antidepressant-like effect possibly through the opioidergic pathway, without inducing tolerance and withdrawal signs (Ghorbanzadeh et al., 2021). Moreover, in the field of ophthalmology, animal studies have shown that Carbamazepine has neuronal protective effects in diabetic retina (Elsherbiny et al., 2019). Therefore, we chose this drug to treat retinal anomalies.

We replicated a rat model of anxiety and depression induced by the CUMS program (Willner et al., 1987). The model is designed to replicate anxiety, depression and other negative emotions seen in humans. Different stimuli mimic the different life experiences people face every day. We used this model to investigate the mechanism by which long-term negative emotions induce retinopathy. After 4 weeks of intervention, the rat body weight, sucrose preference, and field activities decreased. The chronic unpredictable mild stress-induced anxiety and depression-like rat model was established successfully, consistent with previous reports (Liang et al., 2019). Influenced by anxiety and depression, CUMS rats exhibit reduced diet and weight loss, as previously reported (Li et al., 2019; Zhou et al., 2021; Youssef et al., 2022). The weight loss in the CUMS rats was consistent with weight loss during depression in humans (Trief et al., 2014; Fernandez-Mendoza et al., 2015). A decrease in sucrose preference is thought to reflect a decrease in hedonic activity (Wiborg, 2013), which is a key symptom and an effective indicator of depression in animal models (Hill et al., 2012). Open field activity is based on the nature of rodents, which fear open space and wish to explore new environments (Ferreira et al., 2018). The marked decrease in open field activity in rats was consistent with psychomotor hysteresis in patients with depression (Yadid et al., 2000). These behaviors continued until the end of the experiment, reflecting the persistence of anxiety and depression. Human depression is associated with decreased DA (Ferrari and Villa, 2017) and increased CORT (Szymanska et al., 2009). DA is a neurotransmitter responsible for desire in the brain. At 12 weeks, our results showed that serum DA level decreased and CORT level increased in rats, consistent with previous reports (Li et al., 2021). However, MMXY increased the above levels, suggesting that MMXY could effectively improve the weight and behavior abnormalities of CUMS rats, and reverse the reductions in DA and CORT.

The rat retina consists of 10 layers between the sclera and vitreous (Eltony and Abdelhameed, 2017), including RPE, IS/OS, outer limiting membrane, ONL, OPL, INL, IPL, GCL, RNFL and ILM. This 10-layer structure is shown in Figure 5A. OCTA is a non-invasive mode of retinal angiography that can not only analyze the structure of the retina but also shows the network of retinal blood vessels *in vivo* (Arya et al., 2018; Spaide et al., 2018; Coffey et al., 2021). The inner retina is supplied with oxygen and nutrients by the central retinal artery and the outer retina by the choroidal vessels (Kur et al., 2012). Loss of thickness of the inner retina and reduced blood flow is closely related to visual function damage, especially for important structures such as the macula and optic papilla (Yu et al., 2016). It has been reported that retinal vascular density, total retinal blood flow and inner retinal thickness are all reduced in glaucoma patients (Cano et al., 2021). In rats with oxygen-induced retinal injury, retinal thickness and vascular density were reduced (Soetikno et al., 2015). Our OCTA results confirmed that inner retinal blood flow and inner retinal thickness were reduced in CUMS rats, whereas MMXY not only improved retinal blood flow but also restored retinal thickness. On the other hand, Pearson correlation analysis showed that the larger the blood flow area and the higher the blood vessel density of the inner retina, the greater the retinal thickness (Figure 6E).

In addition, MMXY improved the amplitude of ERG OPs2, reflecting increased blood supply in the inner retina (Nagai et al., 2017), consistent with OCTA results (Figures 6C,D,G). HE staining also showed that MMXY improved retinal thickness, consistent with OCTA results (Figures 5B,C,F,G). TEM showed that MMXY recovered retinal ultrastructure. Previous research showed that hypercoagulability is closely associated with retinal diseases (Chen et al., 2021; Tauqeer et al., 2022). In our study, the CUMS rats were in a hypercoagulable state, while MMXY alleviated the hypercoagulable state. We conclude that MMXY improves retinal morphology, retinal thickness and blood flow in CUMS rats.

The ERG is mainly used to reflect the electrophysiological function of the retina (Marmor et al., 2009). The ERG a-wave reflects photoreceptor (cone and rod) function while the b-wave mainly reflects bipolar and Müller cell function (Nagai et al., 2017). In the rat retinal light injury model, the ERG mainly showed a significant decrease in the amplitude of a- and b-waves (Kaidzu et al., 2021). VEPs reflect function of the visual pathway relating to the central visual field (Odom et al., 2010), and are the main objective method to evaluate visual function (Zhang and Yin, 2013). Function is reflected in the peak times and amplitude of N2-P2 (Odom et al., 2016). In the rat optic nerve compression model, the peak times and amplitude of VEPs were prolonged and decreased (Chen et al., 2016). Our results confirm that MMXY can improve the amplitude of a- and b-waves in the scotopic and photopic ERG, as well as N2 and P2 peak time and amplitude in the flash-VEP, suggesting that MMXY can effectively improve the function of retinal cells and of the visual pathway in rats.

The PI3K/Akt/mTOR signaling pathway mainly plays an inhibitory role in the regulation of autophagy (Yang et al., 2019). Autophagy is induced in response to multiple stressors, including starvation, hypoxia, and infection, and fine-tuning this process is important for maintaining homeostasis in cells and tissues (Kaur and Debnath, 2015). However, overactivated autophagy may lead to cell death in the early stages of retinal degenerative diseases (Zhang et al., 2020). Beclin-1 (Kang et al., 2011) and LC3 II/I (Mizushima and Yoshimori, 2007) are indicators reflecting the level of autophagy. In the present study, TEM results showed that autophagosomes were clearly apparent in GCL and INL of the model group, but less apparent in other groups. Results of immunofluorescence showed that Beclin1 and LC3 proteins were highly expressed in the model group, while they were weakly expressed in the control group. Western blot results showed that protein quantification expression of Beclin1 and LC3 increased in the model group, while their expression was lower in the control group. After MMXY treatment, the expression of Beclin1 and LC3 decreased, suggesting that MMXY inhibited autophagy. MMXY increased the expression of phosphorylated proteins in the PI3K-Akt-mTOR signaling pathway, suggesting that this pathway was activated and autophagy inhibition was enhanced, thereby reducing the autophagy level. These results suggest that retinal thinning and abnormal conduction function in CUMS rats may be caused by the enhancement of retinal autophagy through inhibition of the PI3K-AkT-mTOR pathway, while MMXY improved the above situation. Carbamazepine has an antidepressant effect (Post et al., 1986; Ghorbanzadeh et al., 2021), and more importantly, a good neuronal protective effect (Elsherbiny et al., 2019). Our results confirmed Carbamazepine’s protective effect on the retina.

In conclusion, MMXY may be effective in improving retinal morphology and function as well as anxiety and depression-like behaviors in CUMS rats by regulating the PI3K/Akt/mTOR pathway.

## Data Availability

The original contributions presented in the study are included in the article/supplementary material, further inquiries can be directed to the corresponding author.
